# Chromatin accessibility and H3K9me3 landscapes reveal long-term epigenetic effects of fetal-neonatal iron deficiency in rat hippocampus

**DOI:** 10.1186/s12864-024-10230-4

**Published:** 2024-03-21

**Authors:** Shirelle X. Liu, Aarthi Ramakrishnan, Li Shen, Jonathan C. Gewirtz, Michael K. Georgieff, Phu V. Tran

**Affiliations:** 1 Department of Pediatrics, University of Minnesota, Minneapolis, MN 55455 USA; 2 Department of Psychology, University of Minnesota, Minneapolis, MN 55455 USA; 3 Icahn School of Medicine at Mount Sinai, New York, NY 10029 USA

**Keywords:** Iron deficiency, Choline, Chromatin accessibility, H3K9me3 ChIP-seq, Hippocampus, Epigenetics, Transcription

## Abstract

**Background:**

Iron deficiency (ID) during the fetal-neonatal period results in long-term neurodevelopmental impairments associated with pervasive hippocampal gene dysregulation. Prenatal choline supplementation partially normalizes these effects, suggesting an interaction between iron and choline in hippocampal transcriptome regulation. To understand the regulatory mechanisms, we investigated epigenetic marks of genes with altered chromatin accessibility (ATAC-seq) or poised to be repressed (H3K9me3 ChIP-seq) in iron-repleted adult rats having experienced fetal-neonatal ID exposure with or without prenatal choline supplementation.

**Results:**

Fetal-neonatal ID was induced by limiting maternal iron intake from gestational day (G) 2 through postnatal day (P) 7. Half of the pregnant dams were given supplemental choline (5.0 g/kg) from G11–18. This resulted in 4 groups at P65 (Iron-sufficient [IS], Formerly Iron-deficient [FID], IS with choline [ISch], and FID with choline [FIDch]). Hippocampi were collected from P65 iron-repleted male offspring and analyzed for chromatin accessibility and H3K9me3 enrichment. 22% and 24% of differentially transcribed genes in FID- and FIDch-groups, respectively, exhibited significant differences in chromatin accessibility, whereas 1.7% and 13% exhibited significant differences in H3K9me3 enrichment. These changes mapped onto gene networks regulating synaptic plasticity, neuroinflammation, and reward circuits. Motif analysis of differentially modified genomic sites revealed significantly stronger choline effects than early-life ID and identified multiple epigenetically modified transcription factor binding sites.

**Conclusions:**

This study reveals genome-wide, stable epigenetic changes and epigenetically modifiable gene networks associated with specific chromatin marks in the hippocampus, and lays a foundation to further elucidate iron-dependent epigenetic mechanisms that underlie the long-term effects of fetal-neonatal ID, choline, and their interactions.

**Supplementary Information:**

The online version contains supplementary material available at 10.1186/s12864-024-10230-4.

## Background

Iron deficiency (ID) during the gestational and neonatal periods, during which the brain undergoes robust growth [1–3], results in both short- and long-term neurodevelopmental impairments. The acute effects are understandable given the role(s) of iron in neuronal energy metabolism, myelination and neurotransmitter synthesis. The presence of long-term effects in spite of iron treatment in the neonatal period remain unexplained and represent the true cost to society in terms of educational attainment and personal productivity. Extensive longitudinal studies of human cohorts have uncovered a pattern of persistent cognitive impairment and emotional dysregulation following early postnatal ID despite treatment with iron-replacement therapy in childhood [4, 5]. In addition, emerging evidence suggests a significant association between maternal ID and risk of neurodevelopmental disorders such as autism spectrum disorder, attention-deficit hyperactive disorders, and schizophrenia in the offspring [6–9]. Animal models of fetal-neonatal ID exhibit analogous neurodevelopmental impairments and show persistent and widespread gene dysregulation in the adult hippocampus [10–31]. The long-term gene expression changes suggest stable changes in the epigenome as a potential mechanism of action.

The persistence of negative gene expression changes and neurobehavioral phenotypes in preclinical models despite iron treatment commenced in the neonatal period confirms that iron treatment alone is insufficient to completely mitigate the early-life ID effects observed in the clinical populations. Thus, adjunctive therapy is needed for the treatment of early-life ID to rescue the poor long-term neurologic outcomes beyond the already too late iron replacement therapy. In this regard, choline supplementation has been shown to mitigate the deleterious developmental effects of early-life adverse exposures and aberrant genetic factors on brain development [32–35], including fetal alcohol syndrome where brain iron metabolism is perturbed [36, 37]. Choline is an essential nutrient for brain development and can act as a methyl donor to modify DNA and histone methylation [38–43]. In the context of fetal-neonatal ID, choline supplementation has been shown to partially rescue ID-induced abnormal cognitive function and hippocampal transcriptomic and epigenomic changes [12, 24, 25, 44]. The findings suggest an interaction between these two essential nutrients in regulating the brain epigenomic landscape. However, epigenome-wide changes in chromatin accessibility and histone modification due to early-life ID or its treatment with choline supplementation have not been interrogated.

In the present study, we employed Next-Generation Sequencing to assess fetal-neonatal ID-induced global changes in accessible chromatin [45] by Assay for Transposase Accessible Chromatin with sequencing (ATAC-seq). This approach is powered by its unbiased manner to assess chromatin accessibility changes for transcription factor (TF) binding sites. Besides, our previous study demonstrated that early-life ID and prenatal choline altered H3K9me3 enrichment (an index of epigenetic gene silencing [46]) at the iron-dependent histone modifier site [44]. Therefore, in this study we performed a comprehensive investigation of histone H3K9me3 signature at an epigenome-wide level by Chromatin Immunoprecipitation with sequencing (ChIP-seq) and assessed the effects of prenatal choline on these ID-induced epigenetic changes. We hypothesized that fetal-neonatal ID induces changes in chromatin accessibility and H3K9me3 landscapes, which would coincide with, and therefore likely contribute to, the lasting gene dysregulation. Time-targeted prenatal choline-supplementation would normalize multiple early-life ID-induced epigenetic changes. Based on the long-term clinical effects of ID, we predicted that these changes would occur in gene networks regulating neurocognition and emotional behaviors.

## Results

### Fetal-neonatal ID and prenatal choline supplementation alter long-term chromatin accessibility in the adult rat hippocampus

**Fig. 1:**
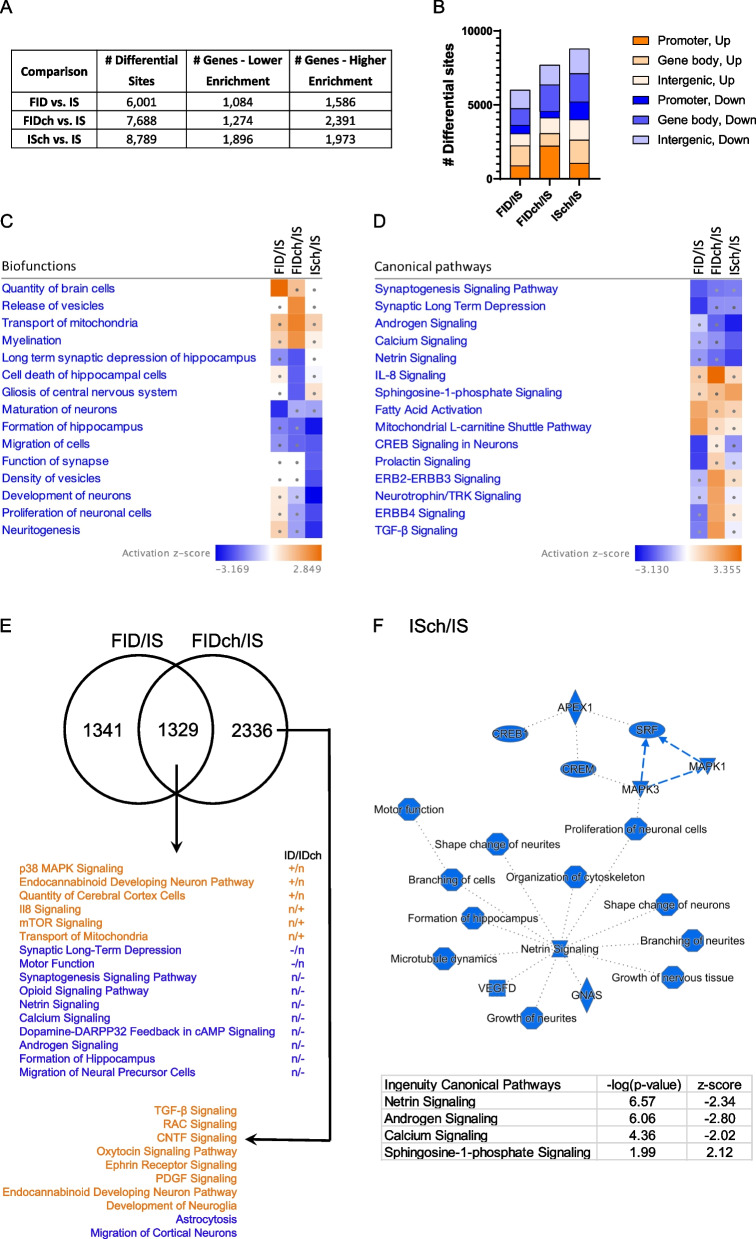
Fetal-neonatal ID and prenatal choline supplementation altered chromatin accessibility landscape in P65 FID rat hippocampus. **A** ATAC-seq data showing differentially-accessible sites and associated genes between treatment groups. Numbers of differential sites include gene bodies, promoter regions (proximal, + 1 K, + 3 K), and intergenic regions. Selection criteria were absolute log2(Fold Change) > 0.2 and false discovery rate *q*-value < 0.05, *n* = 4/group. **B** Distribution of sites with differential accessibility. (C, D) Ingenuity Pathway Analysis (IPA) mapped loci with differential ATAC peaks (within gene bodies or promoter regions) onto biofunctions (**C**) and canonical signaling pathways (**D**), ranked hierarchically. Comparisons were made among formerly iron-deficient (FID), formerly iron-deficient with choline (FIDch), and iron-sufficient with choline (ISch) normalized by the iron-sufficient (IS) control group. Squares with dots have absolute z-scores < 2.0, indicating non-significant findings. Blue and orange colors indicate decreased and increased chromatin accessibilities, respectively. Fewer changes were found in the FID compared to FIDch and ISch groups (absolute z-scores > 2.0). Choline induced both decreased and increased chromatin accessibility in the FIDch and ISch groups. **E** Venn diagram showing overlap and non-overlapping genes modified in FID and FIDch groups. +/−/n indicates increased, decreased, and normalized changes in ATAC signatures. Blue and orange indicate decreased and increased ATAC signatures, respectively. **F** Comparison between ISch and IS control group showing the choline effect on ATAC landscape in adult rat hippocampus. IPA generates a graphical summary showing decreased accessibility in loci regulating hippocampal and cortical development centered on microtubule dynamics and canonical pathways showing both increased and decreased ATAC signatures

To elucidate the long-term changes in genome-wide chromatin accessibility due to fetal-neonatal ID or prenatal choline supplementation, P65 adult rat hippocampus was analyzed by ATAC-seq. Compared to the IS control group, the FID, FIDch or ISch groups showed significant differential sites assigned to known genes with more instances of increased than decreased accessibility (Fig. 1A). Prenatal choline resulted in a higher number of accessible loci in the FIDch compared to the untreated FID group (Fig. 1A, FIDch vs. FID). While these changes occurred across the whole genome, greater proportions occurred within the gene body and the intergenic regions (Fig. 1B).

To define the biological significance of the changes in chromatin accessibility, we employed IPA to functionally annotate the altered loci. Top hierarchically ranked annotated biofunctions showed that fetal-neonatal ID increased accessibility of genes regulating cell proliferation (Fig. 1C, FID/IS) but decreased accessibility of loci regulating neuronal maturation (Fig. 1C, FID/IS). These changes were mitigated by choline treatment (Fig. 1C, FIDch/IS). Choline also increased accessibility of loci implicated in myelination, release of vesicles, and transport of mitochondria and decreased accessibility of genes regulating hippocampal cell death, synaptic long-term depression (LTD) of hippocampus, and gliosis of central nervous system (Fig. 1C, FIDch/IS). Choline treatment of the IS group decreased accessibility of genes regulating hippocampal formation, cell migration, synaptic structures and functions, and neuron proliferation and development (Fig. 1C, ISch/IS).

We next used IPA to annotate accessible loci altered by fetal-neonatal ID or choline treatment onto known canonical pathways. Fetal-neonatal ID results in increased chromatin accessibility of loci in energy metabolism, including fatty acid metabolism and mitochondrial function, but decreased accessibility of loci regulating synaptic signaling (synaptogenesis and LTD) and neurodevelopment (cAMP response element-binding protein [CREB] and prolactin signaling) (Fig. 1D, FID/IS). Choline treatment partially normalized these effects and increased accessibility of loci critical for neuroprotection (e.g., ephrin receptor and neurotrophin/tyrosine receptor kinase [TRK] signaling pathways) and neuroinflammation (interleukin [IL] -8 and transforming growth factor [TGF] -β signaling pathways) in the FIDch group (Fig. 1D, FIDch/IS). In the ISch group, choline treatment increased accessibility of loci in sphingosine-1-phosphate signaling and decreased accessibility of loci in the androgen, calcium and netrin signaling pathways (Fig. 1D, ISch/IS).

To identify gene targets that are epigenetically regulated by both iron and choline, we determined the common loci that were altered in adult FID rats by fetal-neonatal ID and choline. We compared the FID/IS to FIDch/IS ATAC-seq datasets and found that approximately 50% of the loci altered in the FID group were also altered in the FIDch group (Fig. 1E). These common loci mapped onto signaling pathways that regulate synaptic plasticity (mitogen-activated protein kinase [MAPK], calcium, synaptic LTD), energy metabolism (mammalian target of rapamycin [mTOR], transport of mitochondria), neuroinflammation (IL-8, netrin signaling), and reward signaling (endocannabinoid, opioid) (Fig. 1E). Choline treatment not only rescued multiple fetal-neonatal ID effects but also increased (vs. always IS control) accessibility of loci regulating mitochondrial transport, IL-8 and mTOR signaling and decreased accessibility of loci that mapped onto pathways regulating synaptogenesis, hippocampal formation, neuron migration, and opioid signaling (Fig. 1E). 2336 loci showed choline-specific effects with increased accessibility of loci in signaling pathways regulating neuroinflammation (TGF-β, ciliary neurotrophic factor [CNTF], platelet-derived growth factor [PDGF], neuroglia development), synaptogenesis (RAC, ephrin receptor), and social behavior (oxytocin, endocannabinoid) and decreased accessibility of loci regulating astrocytosis and migration of cortical neurons (Fig. 1E).

Our previous transcriptomic study showed significant gene expression changes induced by prenatal choline in the IS adult rat hippocampus [12]. Consistent with these findings, the prenatal choline treated IS (ISch) group exhibited a substantial number of changes in loci accessibility that indicate decreased activity of a functional gene network associated with netrin signaling (Fig. 1F). These changes also showed an increase in sphingosine-1 phosphate signaling but decreases in the androgen and calcium signaling pathways (Fig. 1F, bottom table).

### Prenatal choline increases enrichment of the repressive histone H3K9me3 across the adult rat hippocampal genome

**Fig. 2:**
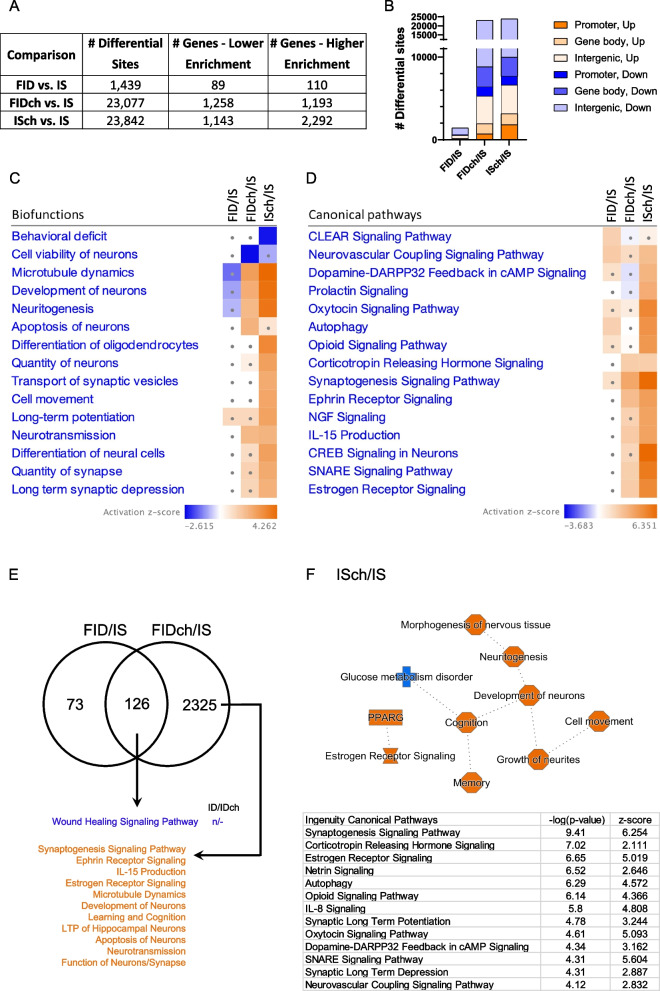
Fetal-neonatal ID and prenatal choline supplementation altered histone H3K9me3 landscape in P65 FID rat hippocampus. **A** H3K9me3 ChIP-seq data showing differential sites and associated genes between treatment groups. Numbers of differential sites include gene bodies, promoter regions (proximal, + 1 K, + 3 K), and intergenic regions. Selection criteria were absolute log2(Fold Change) > 0.2 and false discovery rate *q*-value < 0.05, *n* = 4/group. **B** Distribution of sites with differential H3K9me3 enrichment. (C, D) Ingenuity Pathway Analysis (IPA) mapped differentially-enriched H3K9me3 loci onto biofunctions (**C**) and canonical signaling pathways (**D**), ranked hierarchically. Comparisons were made among formerly iron-deficient (FID), formerly iron-deficient with choline (FIDch), and iron-sufficient with choline (ISch) normalized by the iron-sufficient (IS) control group. Squares with dots have absolute z-scores < 2.0. Blue and orange colors indicate decreased and increased H3K9me3 enrichment, respectively. Early-life ID produced little changes in H3K9me3 signature (absolute z-scores < 2.0). Choline increased H3K9me3 enrichment in the FIDch and ISch groups. **E** Venn diagram showing overlap and non-overlapping genes modified in FID and FIDch groups. +/−/n indicate increased, decreased, and normalized changes in H3K9me3. Blue and orange indicate decreased and increased H3K9me3 signatures. **F** Comparison between ISch and IS control group showing the choline effect on H3K9me3 in adult rat hippocampus. IPA generates a graphical summary showing increased H3K9me3 in loci associated with cognition and estrogen receptor signaling, and canonical pathways showing significantly increased H3K9me3 enrichment

To assess the long-term changes in repressive histone H3K9me3 signature due to either fetal-neonatal ID with or without prenatal choline, we performed an epigenome-wide ChIP-seq analysis of H3K9me3 in the adult P65 rat hippocampus. Compared to the IS control group, the FID group showed 1439 differential sites that were annotated to 199 genes, with 110 showing increased and 89 showing decreased H3K9me3 enrichment (Fig. 2A). Both FIDch and ISch groups showed ~ 23,000 differential sites that mapped onto 2451 and 3435 loci, respectively, based on changes at the promoters and within gene bodies (Fig. 2A). Changes in H3K9me3 enrichment occurred predominantly in the intergenic regions (Fig. 2B).

We then analyzed H3K9me3 changes that mapped onto known genes using IPA. While fetal-neonatal ID alone showed no significant changes of specific biofunctions in the FID group (Fig. 2C, FID/IS), choline treatment exhibited substantial H3K9me3 enrichment changes in the FIDch and ISch groups (Fig. 2C, FIDch/IS and ISch/IS). Enrichment changes in the FIDch group indicate major effects with an increase in loci regulating microtubule dynamics, neurodevelopment, neuritogenesis, neurotransmission, and apoptosis of neurons but a decrease in loci regulating neuronal viability (Fig. 2C, FIDch/IS). The ISch group exhibited even greater H3K9me3 enrichment changes than the FIDch group with an increase at additional loci regulating differentiation of neural cells including oligodendrocytes and synaptic plasticity (e.g., transport of synaptic vesicles, long-term potentiation [LTP], synaptic LTD, quantity of synapse), and a decrease in loci regulating behavioral deficit (Fig. 2C, ISch/IS).

Regarding the canonical signaling pathways, fetal-neonatal ID increased H3K9me3 enrichment at loci regulating coordinated lysosomal expression and regulation (CLEAR), neurovascular coupling, and autophagy pathways (Fig. 2D, FID/IS). Prenatal choline not only rescued the multiple fetal-neonatal ID effects but also increased H3K9me3 enrichment at loci regulating neuroendocrine signaling (corticotropin-releasing hormone, estrogen receptor), synaptic plasticity (synaptogenesis, ephrin receptor, SNARE) and neuroinflammation (IL-15 production) in the FIDch group (Fig. 2D, FIDch/IS). These enrichment changes were choline-specific as the ISch group also showed increased in many of the same pathways along with additional pathways, notably prolactin, oxytocin, nerve growth factor (NGF), and CREB signaling (Fig. 2D, ISch/IS).

To further identify loci and associated biofunctions in adult rats that were altered by both fetal-neonatal ID and choline, we analyzed the overlapping genes in the FID and FIDch datasets. 126 loci were identified and showed decreased enrichment in signaling associated with damaged tissue repair in the FIDch group compared to FID group (Fig. 2E). The non-overlapping 2325 loci in the FIDch group showed higher H3K9me3 enrichment among loci regulating learning and memory (cognition, LTP, neurotransmission), development of neurons (synaptogenesis, microtubule dynamics, apoptosis of neurons, ephrin and estrogen receptor signaling) and IL-15 production (Fig. 2E).

In the ISch group, H3K9me3 enrichment changes indicate overall increases among loci regulating neuronal development (neuritogenesis, cell movement), and social cognition (memory, oxytocin, estrogen, corticotrophin-releasing hormone [CRH]) as well as signaling pathways regulating synaptic transmission (synaptogenesis, synaptic LTD, LTP, SNARE, neurovascular coupling), reward processing (opioid, dopamine-DARPP32 feedback), and neuroinflammation (IL-8) (Fig. 2F).

### Choline rescued fetal-neonatal ID-induced epigenomic changes through distinct regulatory mechanisms

**Fig. 3:**
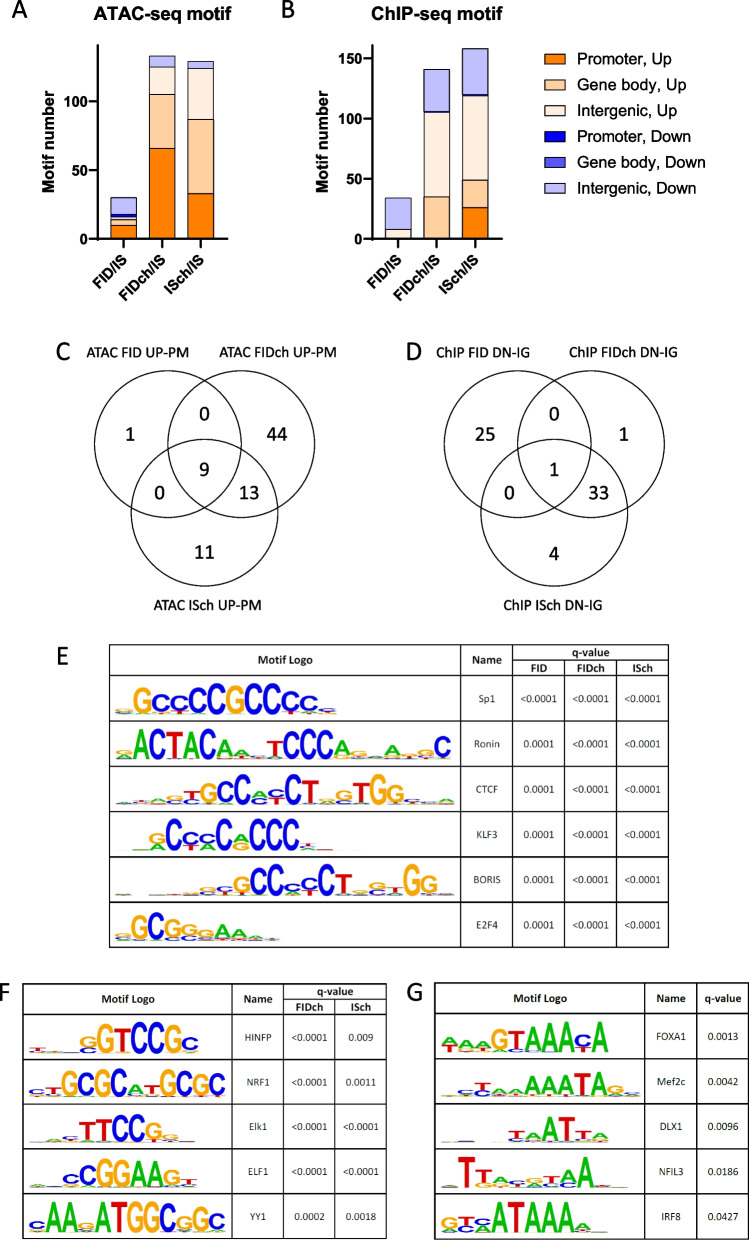
Motif analysis of sites with fetal-neonatal ID-altered chromatin accessibility and H3K9me3 signatures. **A**, **B** Numbers and distributions of motifs identified in each dataset. **C** Overlap of motifs with increased (UP) accessibility in the promoter region (PM) among the 3 experimental groups. **D** Overlap of motifs with decreased (DN) enrichment of H3K9me3 mark the intergenic region (IG) among the 3 experimental groups. **E-G** Representative motifs with increased accessibility in the promoter region shared by FID, FIDch and ISch groups (**E**), only shared by FIDch and ISch groups (**F**), and exclusively in the FIDch group (**G**)

ATAC- and ChIP-seq datasets were analyzed for chromatin accessibility and H3K9me3 enrichment at known TF binding motifs using HOMER motifs analysis in specific genomic regions (i.e., promoter, gene body, intergenic region), to investigate changes in these regulatory mechanisms induced by fetal-neonatal ID and choline supplementation. Compared to the IS group, both fetal-neonatal ID and choline treatment showed more motifs with increased (i.e., open chromatin and H3K9me3 mark) than decreased enrichment (Fig. 3A, B). Chromatin accessibility changes occurred predominantly in motifs located within the promoter and gene body regions, whereas H3K9me3 changes were primarily found in motifs within the intergenic regions (Fig. 3A, B). As expected, very few motifs were overlapped between ATAC- and ChIP-seq datasets, except for those associated with increased enrichment in the promoter region in ISch groups, indicating mutually exclusive actions of the two epigenetic marks (i.e., activation vs. suppression, Supplemental Fig. S1). Most fetal-neonatal ID-increased motif accessibility at promoter regions were not normalized by choline (FIDch, Fig. 3C); similarly, those changes were also induced by choline-supplemented IS rats (ISch, Fig. 3C). In contrast, choline supplementation normalized the fetal-neonatal ID-reduced H3K9me3 enrichment in motifs found within the intergenic regions (FIDch, Fig. 3D). Such effects were driven primarily by choline as shown by a major overlap of motifs between the choline-treated groups (ISch vs FIDch, Fig. 3D, Supplemental Fig. S2).

Since the functions of TF binding motifs were best characterized at the promoter region, we focused the functional analysis of these specific motifs. Common motifs among the 3 experimental groups showed increased accessibility (ATAC) for TFs involved in mitochondrial function (RONIN) and neurite outgrowth and neural development (SP1/KLF, E2F, CTCF, BORIS) (Fig. 3C, E; Supplemental Figs. S3-5). Choline-specific effect regardless of fetal-neonatal iron status includes increased accessibility of TFs involved in neurodegeneration (HINFP), oxidative phosphorylation (NRF1), and CNS development (ELF1, YY1, ELK) (Fig. 3F; Supplemental Fig. S4–5). The choline-supplemented FID group (FIDch) showed increased accessibility of TFs involved in neuroinflammation (IRF, NFIL3) and the development of GABAergic and dopaminergic neuron (FOXA1, DLX) (Fig. 3G, Supplemental Fig. S4). One member from the IRF family (IRF2) also showed increased accessibility in the ISch group (*q*-value = 0.0017, Supplemental Fig. S5).

### Integration of RNA-seq, ATAC-seq, and ChIP-H3K9me3-seq data revealed an increase of synaptic transmission in adult rat hippocampus due to early-life ID or prenatal choline supplementation

**Fig. 4:**
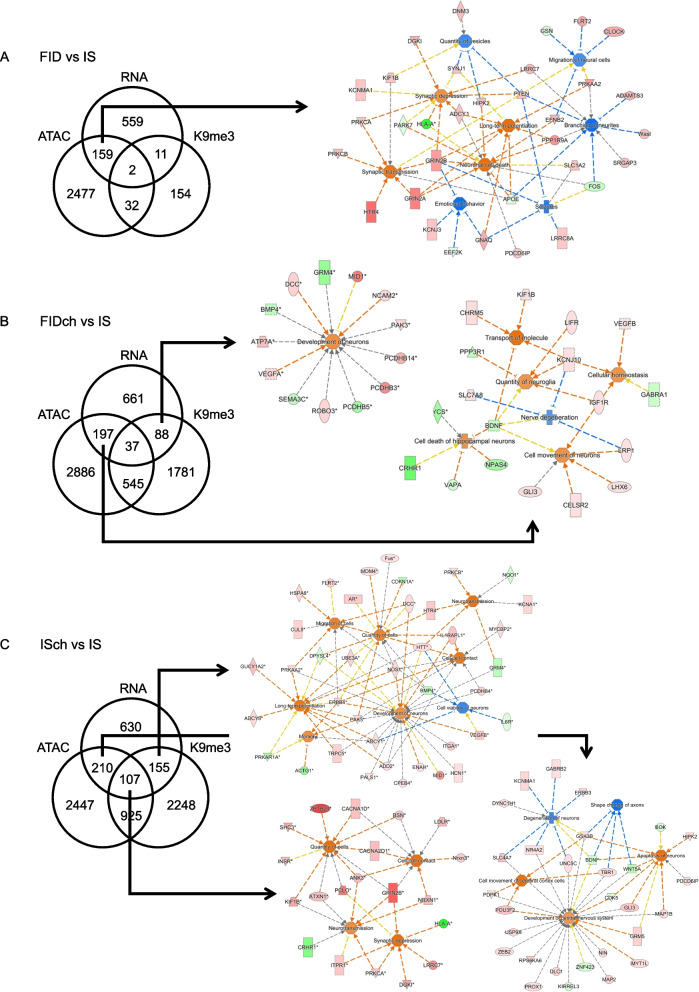
Integration of P65 hippocampal RNA-seq, ATAC-seq, and ChIP-H3K9me3-seq data. **A** FID and IS comparison showing 159 of 731 (22%) loci with specific changes in chromatin accessibility. These changes indicate activated synaptic transmission and neuronal cell death accompanied by inhibited branching of neurites, migration of neural cells, and emotional behavior. **B** FIDch vs IS comparison showing 88 loci with specific H3K9me3 changes, which indicate an activated neuronal development. 197 loci with specific changes in chromatin accessibility indicate activated cellular function, including cellular homeostasis, cell movement, cell death, and quantity of neuroglia, but inhibited nerve degeneration. **C** ISch and IS comparison showing 107 loci with both ATAC and H3K9me3 changes, which indicate activated synaptic transmission and quantity of cells. 155 loci with specific H3K9me3 changes indicate reduced neuronal viability but activated development of neurons, neurotransmission, and cell migration. 210 loci with specific changes in chromatin accessibility indicate both inhibited degeneration and activated apoptosis of neurons via different set of genes, as well as activated development of the nervous system

Congruency of transcriptomic and epigenomic signatures was determined between each treatment (FID, FIDch or ISch) and control (IS) group to further elucidate altered biofunctions and canonical pathways. In the FID group, 159 (22%) loci showed changes in both expression and chromatin accessibility, indicating increased synaptic functions (synaptic transmission, synaptic depression, and LTP) and decreased neural cell migration, neurite branching, and emotional behaviors (Fig. 4A). In the FIDch group, 197 (24%) and 88 (13%) differentially expressed (DE) genes also showed change in chromatin accessibility and H3K9me3, respectively (Fig. 4B). These changes indicate an increased in neurodevelopment, cell movement, neuroglia quantity, and cellular functions (Fig. 4B). In the ISch group, 210 (29%) and 155 (24%) DE genes showed changes in chromatin accessibility and H3K9me3, respectively, that indicate reduced neural and axonal development and enhanced cell migration, synaptic transmission, and memory (Fig. 4C). The 107 common loci that were changed in both ATAC and H3K9me3 from the ISch group showed expression changes indicating increased cell quantity and synaptic transmission (Fig. 4C).

**Fig. 5:**
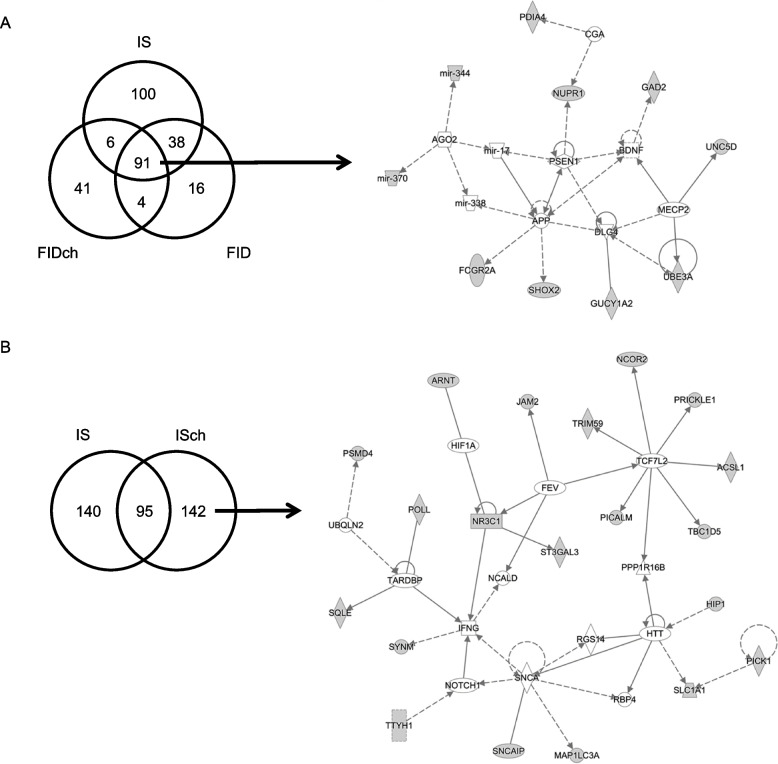
Alignments of ATAC-seq and H3K9me3-seq peaks among treatment groups. **A** Overlapping ATAC and H3K9me3 peaks among IS, FID, and FIDch groups localized to the intergenic regions of 91 nearby genes, which mapped onto a gene network centering on the Brain-derived neurotrophic factor (BDNF), Presenilin-1 (PSEN1), and Amyloid precursor protein (APP), and methylcytosine-binding protein 2 (MeCP2). **B** Overlapping ATAC and H3K9me3 peaks between IS and ISch groups showed 142 choline-specific effect genes that mapped onto a gene network centering on alpha-synuclein (SNCA), Huntingtin (HTT), transcription factor 7 like 2 (TCF7L2), and interferon gamma (INFG)

Since ATAC and H3K9me3 peaks showed overlap mainly in the intergenic regions across all four experimental groups (Table 1), we analyzed the nearest genes to these overlapping regions and identified 91 common genes among IS, FID and FIDch groups. These genes mapped onto a network centering on brain-derived neurotrophic factor (BDNF), presenilin-1 (PSEN1), and amyloid precursor protein (APP) (Fig. 5A). The choline-specific effect showed 142 genes that mapped onto a network centering on alpha-synuclein (SNCA), Huntingtin (HTT), transcription factor 7 like 2 (TCF7L2), and interferon gamma (INFG) (Fig. 5B).

**Table 1:** Overlapping regions between ATAC and H3K9me3 peaks. The overlapping peaks between the two datasets were primarily located in intergenic regions

Treatment Group	# Overlapping Regions	# Nearest Loci	IPA-Annotated Diseases and Functions
*IS*	868	235	Development of CNS, Long-Term Potentiation (LTP)
*ISch*	593	237	Synaptic transmission of cerebrocortical cells, LTP of hippocampal neurons
*FID*	551	149	Differentiation of neurons, Migration of cells, LTP
*FIDch*	563	142	Cerebral disorder, Tauopathy, LTP, Migration of neuroglia

## Discusion

Early-life ID and prenatal choline produce persistent and widespread hippocampal gene dysregulation in preclinical models [11, 12, 47]. We previously demonstrated DNA methylation changes in the developing iron-deficient hippocampus [48] and chromatin methylation changes at the *Bdnf* gene in the FID hippocampus [12], providing evidence for acute and persistent epigenetic modifications induced by early-life ID. To systematically determine the stable long-term epigenetic changes induced by early-life ID in the iron-repleted adult hippocampus, the present study assessed and integrated the landscapes of accessible chromatin modifications (ATAC vs. H3K9me3) and transcriptomic changes from the FID rat hippocampus. The findings reveal that fetal-neonatal ID altered chromatin accessibility accounting for about 22% of gene expression changes, particularly among loci that regulate neurite growth and synaptic transmission; however, fetal-neonatal ID had little (~ 1.7%) effect on the repressive histone H3K9me3 mark. These lower overlap percentages might partially be due to sampling from separate groups of animals, but could also indicate other mechanisms underlying the long-term gene expression changes, suggesting an involvement of other epigenetic modifications. Thus, integration of additional epigenetic marks (e.g., H3K27me3, H3K4me3, 5mC, and 5hmC [49, 50]) is needed in future studies to more fully elucidate epigenetic mechanisms contributing to the fetal-neonatal ID-induced long-term gene dysregulation.

We addressed the prenatal choline effects on specific epigenetic modifications given its initial identification as a potential adjunctive treatment for early-life ID [25, 51]. Choline partially rescued the fetal-neonatal ID effects, accounting for 24% of gene expression changes. Interestingly, prenatal choline had a significant effect on both ATAC and H3K9me3 changes in the IS group, particularly among loci regulating neurotransmission and opioid signaling, suggesting either excessive dosing or duration of choline treatment. Thus, more studies assessing choline dose-responses are needed in order to identify appropriate dosing for its proposed used as an adjunct therapy for early-life ID anemia [52]. Collectively, these findings reveal a lasting consequence of early-life ID and prenatal choline on the adult male rat hippocampal epigenome.

Fetal-neonatal ID resulted in more loci showing increased than decreased accessibility, consistent with our transcriptomic data. These findings support the conclusion that changes in ATAC partially account for the long-term epigenetic modifications induced by early-life ID [53]. Nevertheless, it is not surprising that the reduced chromatin accessibility occurred among loci regulating maturation of hippocampal neurons, which may account for the impaired cognitive function exhibited by FID rats [24, 25]. Our group recently reported the effect of phlebotomy-induced ID anemia on neuroinflammation [54]. Given the important role of microglia in neural development and function [55], the long-term ID-induced epigenetic changes in loci regulating neuroinflammation signaling pathways could contribute to the abnormal cognition, emotional behavior, and neurodevelopmental disorders (e.g., autism, schizophrenia, Alzheimer disease) observed in the FID adult animals [21, 26, 29].

The established beneficial effects of prenatal choline demonstrated by others in preclinical models of other neurodevelopmental conditions [33–35, 41], by us in this model of fetal-neonatal ID [12, 51] and clinically in children [32], as well as choline’s role as a methyl donor [56] justified the rationale for assessing its long-term effects on the neuroepigenome. A major finding was of long-term choline effects on both ATAC and H3K9me3 landscapes in the hippocampus of the FID and IS control groups. In the FID group, choline supplementation not only normalized fetal-neonatal ID-reduced accessibility of genes that regulate neuronal maturation and synaptic plasticity (e.g., synaptogenesis, LTD, CREB signaling pathways) but also promoted accessibility of genes regulating myelination, mitochondrial transport, and synaptic function (ephrin and neurotrophin signaling). These effects could account for the partial recovery of hippocampal-dependent learning and memory function in FIDch adult rats [51]. While these long-term epigenetic changes were found in the hippocampus, a focus of our study, they could also occur in the cerebral cortex and cerebellum as these brain structures have a long developmentally-sensitive window [57]. Future studies could investigate the epigenomic effects of early-life ID on other developing brain regions as we have in a previous study [58].

A novel and unique finding of prenatal choline effect in the IS group (ISch) was the increased H3K9me3 enrichment among loci regulating opioid and dopamine-DARPP32 signaling pathways, suggesting that choline can influence the reward circuitry potentially involving the hippocampal-cortical-nucleus accumbens connectivity [59, 60]. This finding raises possible concerns as to whether prenatal choline may affect the reward signaling circuity in adulthood given the chromatin changes in loci implicating altered balance of dopaminergic, glutamatergic, and GABAergic synaptic transmission [61–64]. Thus, the use of choline as a therapeutic agent will need more rigor scrutiny to determine the appropriate dose and duration during gestation so as to maximize the beneficial effects while minimizing potential negative impacts.

The fetal-neonatal ID or choline effects on ATAC and H3K9me3 signatures were largely distinct with limited overlapping regions, indicating that the majority of peaks between ATAC and H3K9me3 were mutually exclusive as expected given that they generally have opposing effects (activation vs. repression) on gene regulation [45, 46]. However, the genomic regions showing both ATAC and H3K9me3 changes by early-life ID were found in a gene network that include BDNF, APP, and PSEN1, which we have documented previously using transcriptomic and targeted gene expression analyses [21, 47]. The findings highlight convergent epigenetic modifications as well as reveal potential long-distance regulatory elements for specific gene networks that can be dysregulated by early-life ID. Likewise, prenatal choline produced convergent epigenetic modifications within a specific gene network regulating factors that contribute to neurological diseases, including SNCA, HTT, and NR3C1. Thus, it is important to determine the long-term neurological effects of prenatal choline supplementation to fully evaluate its beneficial effects beyond cognitive function. Lastly, analysis of fetal-neonatal ID and choline effects on TF binding motifs reveal that choline may rescue transcriptional and behavioral abnormalities induced by early-life ID using molecular machineries distinct from those directly affected by fetal-neonatal ID. TFs that were implicated only in the FIDch group such as FOXA1, DLX and certain members from the IRF family may serve as potential targets for the development of an interventional strategy to prevent long-term neurologic outcomes.

There are two limitations of our study. First, to keep in line with our prior behavioral analyses of this model, the study design involved culling litters and using a single male rat from each litter for the performed experiments. This was to minimize the effects associated with litter size and maternal nursing behavior. As such, this design potentially increases the variation of inherent epigenomes within the same experimental group, which could lead to smaller signal-to-noise ratio as epigenetic marks are shown to be heritable [65] and varied across litters. This inherent cross-litter variation in could pose a risk to the reproducibility of the study. Second, despite a statistical power of ~ 70%, our experimental sample size (*n* = 4/group), while similar to multiple published epigenetic studies [49, 66–70], could be underpowered to small differences between groups. Future studies could increase the sample size to 6/group, which would increase the estimated power to > 80%, to detect these small changes.

## Conclusions

Early-life ID produces lasting and widespread changes in the adult iron-repleted rat hippocampal epigenome, providing insights into molecular mechanisms mediating the long-term gene dysregulations. This study provides evidence that changes in chromatin accessibility could partially account for this persistent gene dysregulation in adulthood. The mechanisms underlying early-life ID-altered chromatin landscapes were likely independent of modifiers of H3K9me3 mark such as the iron-containing KDM4 histone demethylase and the G9A methyltransferase. Conversely, choline-mediated epigenetic changes in the rat hippocampus involved modification of the H3K9me3 landscape, implicating H3K9 methyltransferases and demethylases. Since epigenetic regulation involves complex interactions among iron-dependent and -independent factors, this study provides a foundation for further research to fully investigate the relative contributions of others epigenetic modifications underlying early-life ID-induced gene dysregulation and to identify potential therapeutic targets for the prevention of long-term adverse outcomes of early-life ID.

## Methods

### Animals

Gestational day (G) 2 pregnant Sprague-Dawley rats were purchased from Charles River Laboratories (Wilmington, MA). Rats were maintained on a 12-hr:12-hr light/dark cycle with ad lib food and water. Fetal-neonatal ID was induced by dietary manipulation as previously described [25, 71] to induce a degree of ID that occurs in human newborns [72]. In brief, pregnant rats were given a purified iron-deficient diet (4 mg Fe/kg, TD 80396, Harlan Teklad, Madison, WI) from G2 to postnatal day (P) 7 and a purified iron-sufficient diet (200 mg Fe/kg, TD 09256, Harlan Teklad) thereafter. Both diets were similar in all respects except for their ferric citrate content. Control iron-sufficient rats were generated from pregnant rats maintained on the iron-sufficient diet. Half of the dams on iron-sufficient or iron-deficient diet received dietary choline supplementation (5.0 g/kg choline chloride ([73, 74], Iron-sufficient + choline: TD 1448261, Iron-deficient + choline: TD 110139) from G11–18, while the remaining dams received iron-modified diets with standard choline content (1.1 g/kg). Thus, dams and their litters were randomly assigned to the following groups: iron-deficient with choline supplementation, iron-deficient without choline supplementation, iron-sufficient with choline supplementation, and iron-sufficient without choline supplementation. All litters were culled to 8 pups at birth with equal numbers of male and female pups to minimize the effects associated with sex and litter size in maternal nursing behavior. Only male offspring were used in the current study. To avoid litter-specific effects, tissue from a single male rat from each litter was used in each experiment. Rats were weaned on P21 and maintained on an iron-sufficient diet thereafter. The pups were studied at P65 after complete brain iron repletion [75]. Therefore, the final study groups were formerly iron-deficient without choline supplementation (FID), formerly iron-deficient with choline supplementation (FIDch), iron-sufficient with choline supplementation (ISch), and always iron-sufficient without choline supplementation (IS, control group). The University of Minnesota Institutional Animal Care and Use Committee approved all experiments in this study (Protocol # 2001-37802A).

### Hippocampal dissection

P65 rats were euthanized by injection of pentobarbital (100 mg/kg, intraperitoneal) followed by decapitation. Brains were removed and bisected along the midline on an ice-cold metal block. The hippocampus was isolated, flash-frozen in liquid nitrogen, and stored at − 80 °C.

### Nuclei isolation and DNA transposition

Nuclei isolation and DNA transposition were carried out as previously described [76] with modifications. Briefly, flash-frozen hippocampus was thawed in cold homogenization buffer (5 mM CaCl_2_, 3 mM Mg(AC)_2_, 10 mM Tris pH 7.8, 0.0167 mM PMSF, 0.167 mM β-mercaptoethanol, 1x proteinase inhibitor (cOmplete), 320 mM sucrose, 0.1 mM EDTA, 0.1% CA630), and homogenized using a Pellet Pestle motor and syringe. Equal volume of 50% iodixanol solution (5 mM CaCl_2_, 3 mM Mg(AC)_2_, 10 mM Tris pH 7.8, 0.017 mM PMSF, 0.17 mM β-mercaptoethanol, 1x proteinase inhibitor (cOmplete), 50% iodixanol) was added to homogenate to reach a final concentration of 25% iodixanol. 29% (5 mM CaCl_2_, 3 mM Mg(AC)_2_, 10 mM Tris pH 7.8, 0.017 mM PMSF, 0.17 mM β-mercaptoethanol, 1x proteinase inhibitor (cOmplete), 160 mM sucrose, 29% iodixanol) and 35% (5 mM CaCl_2_, 3 mM Mg(AC)_2_, 10 mM Tris pH 7.8, 0.017 mM PMSF, 0.17 mM β-mercaptoethanol, 1x proteinase inhibitor (cOmplete), 160 mM sucrose, 35% iodixanol) iodixanol solution were sequentially added under the 25% iodixanol solution layer. The 3-layer system was centrifuged in a swinging bucket centrifuge at 4255 x g for 20 min. After centrifugation, nuclei were isolated and collected from the 29–35% iodixanol solution interface. Isolated nuclei were counted with trypan blue (0.4%) and 50,000 nuclei were transferred into a tube containing 1 ml ATAC-RSB (10 mM Tris pH 7.8, 10 mM NaCl, 3 mM MgCl_2_) with 0.1% Tween-20. Nuclei were pelleted by centrifugation at 500 x g for 10 min. All steps above were conducted on ice or at 4 °C. Nuclei were resuspended in transposition mix (25 μL 2x TD buffer (Illumina), 16.5 μL PBS, 0.5 μL 10% Tween-20, 1% digitonin, 2.5 μL Tn5 transposase (Illumina), 5 μL H_2_O), and incubated at 37 °C for 30 min in a thermomixer at 1000 rpm. Transposed DNA was purified using MinElute Reaction Cleanup kit (Qiagen).

### Chromatin immunoprecipitation (ChIP)

ChIP experiments were performed as previously described [77] with modifications. In brief, chromatin was prepared from hippocampal samples following the manufacturer’s recommendations (Millipore, Temecula, CA). Samples were homogenized in ice-cold PBS (500 μL) using a Pellet Pestle motor and pelleted by centrifugation at 13 K rpm (30s). The pellets were resuspended in PBS and cross-linked in 1% formaldehyde solution (Sigma). Following the removal of fixative and PBS rinses, lysates were resuspended in 500 μL lysis buffer (1% SDS, 10 mM EDTA, 50 mM Tris pH 8.1, 1 mM PMSF, 10 μL 10X protease inhibitor cocktails (Roche, Indianapolis, IN)), incubated in an ice bath for 10 min, and sonicated (Bioruptor Pico, Diagenode) to shear DNA. Sonicated lysates were diluted 10-fold with ChIP dilution buffer (0.01% SDS, 1.1% Triton X-100, 1.2 mM EDTA, 16.7 mM Tris-pH 8.1, 167 mM NaCl) and pre-cleared with 75 μL of Protein A agarose (50% slurry, Sigma). Pre-cleared chromatin lysate was immunoprecipitated by a ChIP-grade rabbit polyclonal antibody against histone H3K9me3 (Cat. # C15410193, Diagenode) with end-over-end rotation (4 °C, overnight). The antibody-histone complex was collected through the addition of 75 μL Protein A agarose slurry with mixing (4 °C, ≥ 1 hour). Following washes (per manufacturer’s protocol, Millipore), the immune-histone complex was eluted in 500 μL of elution buffer (1% SDS, 0.1 M NaHCO_3_). Reverse cross-linking was achieved by incubation in NaCl (0.2 M, 65 °C, overnight). Protease digestion (20 μg proteinase K, 20 mM EDTA, 100 mM Tris-pH 6.5, 45 °C, 1 hour) was performed to recover DNA, which was further purified using phenol/chloroform extraction and EtOH precipitation (1/10 volume 3 M sodium acetate, pH 5.2, 2 volume ethanol). Levels of enriched *Gapdh* (active) and *Myod1* (inactive) loci were used to validate ChIP experiments by real-time PCR.

### Real-time PCR

For validation analysis of precipitated DNA from ChIP experiments, SYBR-green PCR (Fast SYBR green master mix, ABI) was used to amplify *Gapdh and Myod1*. Input DNA (10%) was used as a normalizer to account for input amount (ΔCt). Real-time PCR was performed with QuantStudio™ 5 (Thermo Fisher).

### Next-generation sequencing

Recovered DNA from ATAC or ChIP was delivered to the University of Minnesota Genomics Center for quality control, library preparation and sequencing. DNA was first quantified using the PicoGreen dsDNA Assay Kit (Invitrogen). ATAC-seq library preparation was performed in accordance with the ATAC-seq protocol as described by Buenrostro et al. [78]. Library preparation for ChIP-seq was completed using ThruPLEX® DNA-Seq (Rubicon Genomics) kit. Libraries were assessed for quality using electrophoresis on an Agilent Bioanalyzer (Agilent) and size-selected for 200 ~ 800 bp fragments. Selected libraries were quantified again, and sequencing was performed at a depth of ~ 46 million reads per sample using NovaSeq 6000 for ATAC-seq, and a depth of ~ 25 million reads per sample using HiSeq 2500 for ChIP-seq to generate 50-bp pair-end reads.

### Bioinformatics

ChIP-seq and ATAC-seq datasets, comprising 16 samples with 4 biological replicates in each condition (IS, ISch, FID, and FIDch) were aligned to the Ensembl v90 rat reference genome (rn6) using HISAT2 (v2.2.1) [79]. MACS (v2.1.0) was utilized to assess sites of chromatin accessibility in ATAC-seq samples and sites of H3K9me3 histone modification (peaks) for each of the 4 conditions [80]. MACS2 was run with parameters --bw 300 -p 1e-05 for the ChIP-seq data and -p 1e-05 --nomodel --shift 100 --extsize 200 for ATAC-seq data. diffReps [81] was utilized to find differential sites for the comparisons to the IS control group (FID, FIDch, or ISch vs. IS). diffReps was run with default parameters. Global analysis of overlapping between ATAC and H3K9me3 peaks was performed using BEDTools (PMID: 20110278) [82] with 1 bp overlapping threshold to identify regions modified by both markers. Differential sites were mapped to genes using the tool HOMER (PMID: 20513432) [83]. Visualization of alignments of ATAC and H3K9me3 peaks for representative genes *Bmp3*, *Tnks*, and *Ireb2* was performed using Integrative Genomics Viewer (Supplemental Fig. S6).

For RNA-seq data, datasets from a previous study [12] were reprocessed using FastQC and HISAT2 (v2.1.0). Gene quantification was done via featureCounts (PMID: 24227677) [84] to obtain the raw counts of reads mapping to genes. Differentially expressed genes (absolute log2(Fold Change) > 0.2 and *p*-value < 0.05) were identified using the edgeR (PMID: 19910308) [85] package in CLC Genomics Workbench software (Qiagen, Redwood City, CA) with a negative binomial generalized log-linear model fit to the raw read counts. Each experimental group (FID, FIDch or ISch) was compared to the IS control group. Parameter fitting was performed separately for each pairwise comparison. RNA-seq results were previously validated using real time PCR [12].

### Ingenuity pathway analysis (IPA)

Differentially modified genes from pairwise comparisons between treatment and IS control group (e.g., FID vs. IS, FIDch vs. IS, and ISch vs. IS) were analyzed by IPA (Qiagen), a knowledge-based database of > 8 million findings, to identify pertinent pathways, gene networks, and biofunctions. Data were analyzed using core analysis with Fisher’s exact test *p*-value ≤0.05 and absolute Z-score ≥ 2.0. Positive and negative Z-scores indicate increased and decreased activity, respectively.

### HOMER motif analysis

The program ‘findMotifsGenome.pl’ from the tool HOMER (PMID: 20513432) [83] was utilized to identify motifs of transcription factors among differential sites identified by diffReps (PMID: 23762400) [81] after applying an absolute log2(Fold Change) cutoff of 0.2 and an adjusted *p*-value cutoff of 0.05 [83].

### Supplementary Information

**Supplementary Material 1.**

**Supplementary Material 2.**

**Supplementary Material 3.**

**Supplementary Material 4.**

**Supplementary Material 5.**

**Supplementary Material 6.**

## Data Availability

The datasets generated and/or analysed during the current study are available in the Gene Expression Omnibus repository, GSE229266, GSE244208 (reviewer access token: mrepmwoebrwttsl).

## Abbreviations

APP: Amyloid precursor protein
ATAC: Assay for transposase-accessible chromatin
BDNF: Brain-derived neurotrophic factor
ChIP: Chromatin immunoprecipitation
CLEAR: Coordinated lysosomal expression and regulation
CNTF: Ciliary neurotrophic factor
CREB: cAMP response element-binding protein
CRH: Corticotrophin-releasing hormone
DE: Differentially expressed
FID: Formerly iron-deficient
FIDch: Formerly iron-deficient with choline
G: Gestational day
ID: Iron deficiency
IL: Interleukin
IPA: Ingenuity pathway analysis
IS: Iron-sufficient
ISch: Iron-sufficient with choline
LTD: Long-term depression
LTP: Long-term potentiation
MAPK: Mitogen-activated protein kinase
mTOR: Mammalian target of rapamycin
NGF: Nerve growth factor
P: Postnatal day
PDGF: Platelet-derived growth factor
PSEN: Presenilin
TF: Transcription factor
TGF: Transforming growth factor
TRK: Tyrosine receptor kinase
