# Supplementary material for: Chromatin accessibility and H3K9me3 landscapes reveal long-term epigenetic effects of fetal-neonatal iron deficiency in rat hippocampus
Source: BMC Genomics. 2024 Mar 21;25:301. doi: 10.1186/s12864-024-10230-4 (PMC10956188; doi:10.1186/s12864-024-10230-4)
Supplement: Supplementary file 1 — Supplementary Material 1. [file 12864_2024_10230_MOESM1_ESM.pdf]

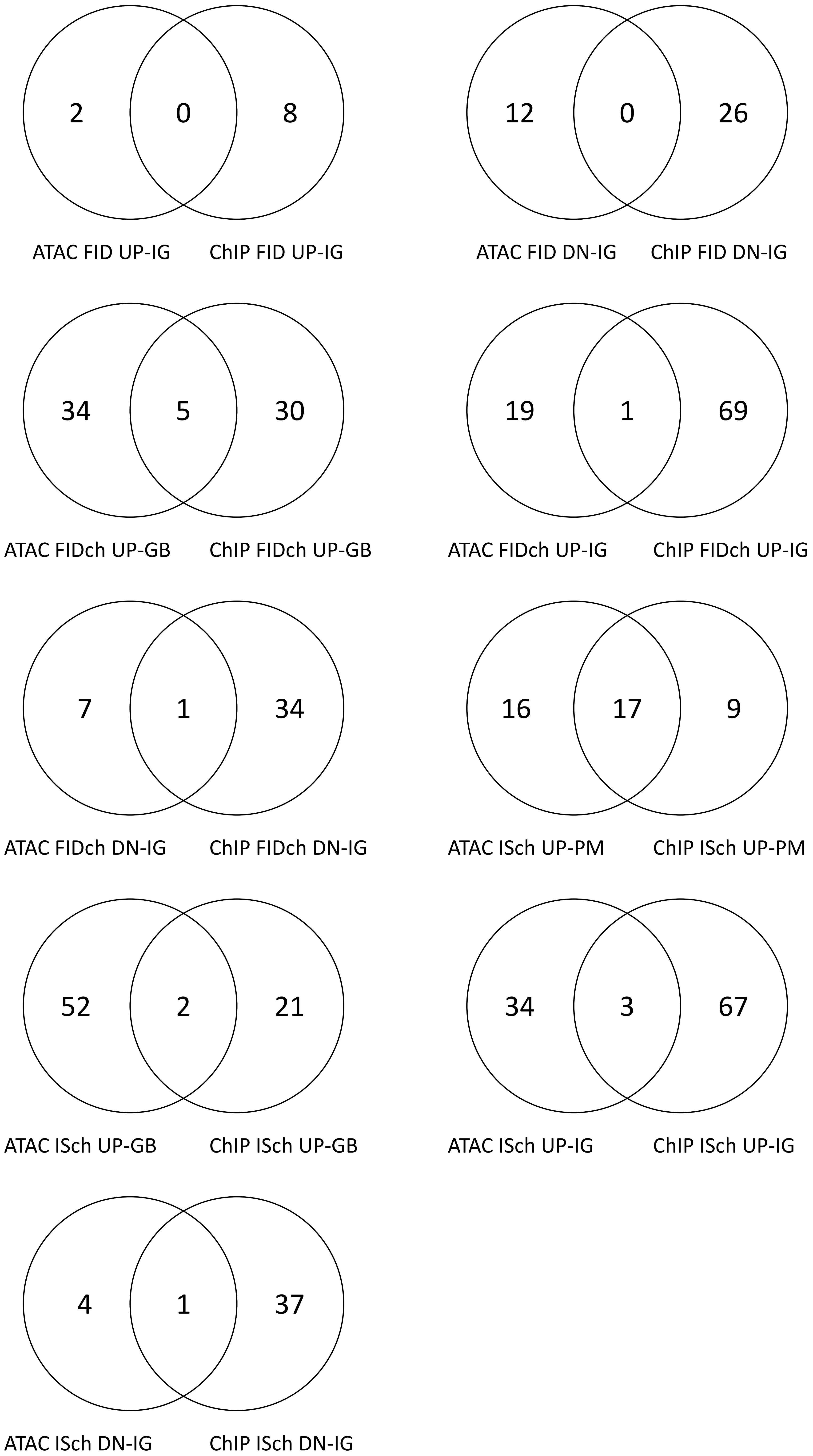

S1: Overlap of motifs between ATAC- and H3K9me3 ChIP-seq. IG, intergenic region; GB, gene body; PM, promoter.
