# Supplementary material for: Chromatin accessibility and H3K9me3 landscapes reveal long-term epigenetic effects of fetal-neonatal iron deficiency in rat hippocampus
Source: BMC Genomics. 2024 Mar 21;25:301. doi: 10.1186/s12864-024-10230-4 (PMC10956188; doi:10.1186/s12864-024-10230-4)
Supplement: Supplementary file 2 — Supplementary Material 2. [file 12864_2024_10230_MOESM2_ESM.pdf]

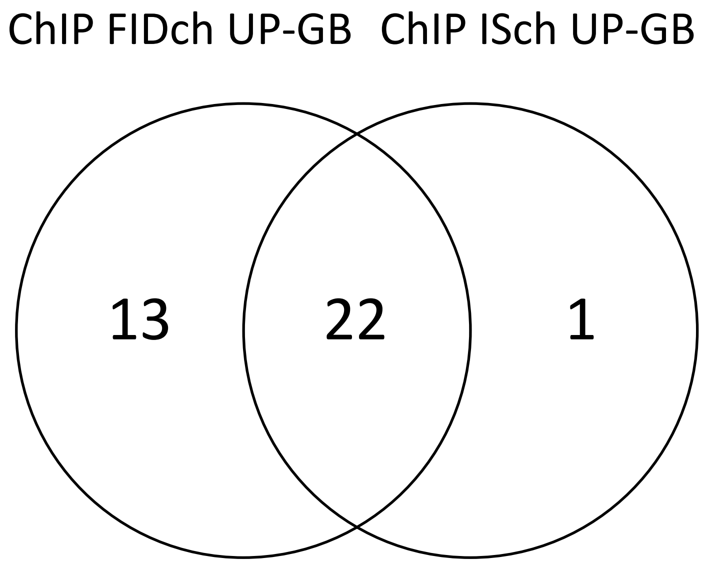

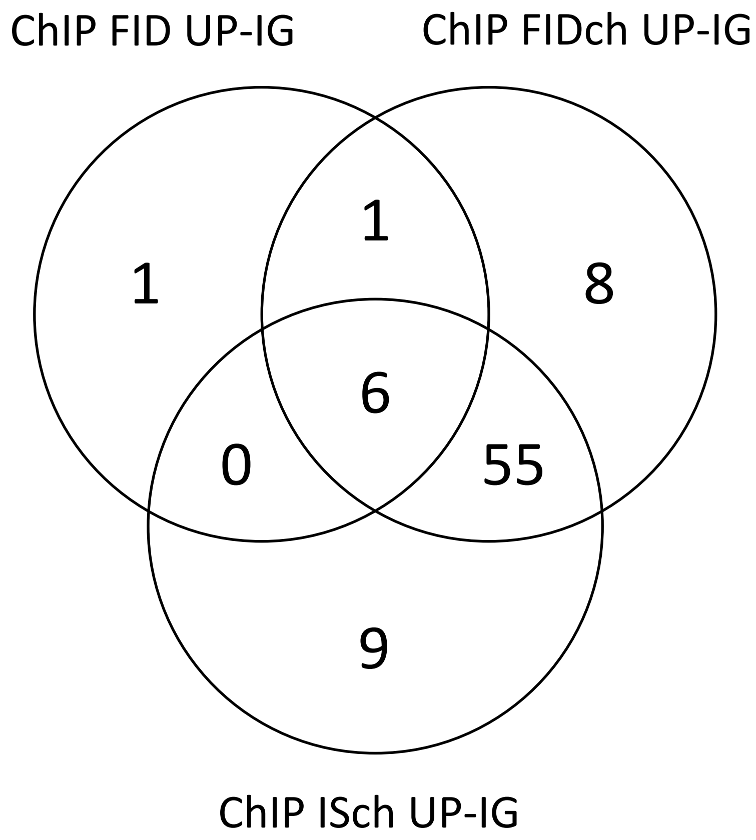

S2: Overlap of motifs with increased H3K9me3 enrichment among experimental groups. GB, gene body; IG, intergenic region.
