# Supplementary material for: Chromatin accessibility and H3K9me3 landscapes reveal long-term epigenetic effects of fetal-neonatal iron deficiency in rat hippocampus
Source: BMC Genomics. 2024 Mar 21;25:301. doi: 10.1186/s12864-024-10230-4 (PMC10956188; doi:10.1186/s12864-024-10230-4)
Supplement: Supplementary file 3 — Supplementary Material 3. [file 12864_2024_10230_MOESM3_ESM.pdf]

S3 Motifs identified by ATAC-seq and HOMER analysis in the promoter region with increased accessibility in the FID group.

Homer Known Motif Enrichment Results (ID_uppm.tab_genome)

Homer *de novo* Motif Results
Gene Ontology Enrichment Results
Known Motif Enrichment Results (txt file)
Total Target Sequences = 885, Total Background Sequences = 44535

Rank	Motif	Name	P-value	log P-pvalue	q-value (Benjamini)	# Target Sequences with Motif	% of Targets Sequences with Motif	# Background Sequences with Motif	% of Background Sequences with Motif	Motif File	SVG
1	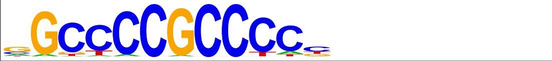	Sp1(Zf)/Promoter/Homer	1e-9	-2.143e+01	0.0000	189.0	21.36%	6132.5	13.77%	motif file (matrix)	svg
2	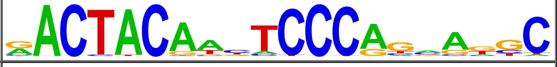	Ronin(THAP)/ES-Thap11-ChIP-Seq(GSE51522)/Homer	1e-6	-1.517e+01	0.0001	33.0	3.73%	596.3	1.34%	motif file (matrix)	svg
3	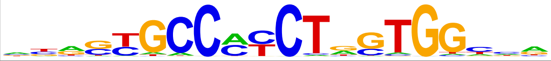	CTCF(Zf)/CD4+-CTCF-ChIP-Seq(Barski_et_al.)/Homer	1e-6	-1.513e+01	0.0001	140.0	15.82%	4590.7	10.31%	motif file (matrix)	svg
4	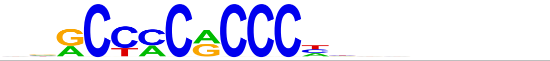	KLF3(Zf)/MEF-Klf3-ChIP-Seq(GSE44748)/Homer	1e-6	-1.508e+01	0.0001	314.0	35.48%	12346.6	27.71%	motif file (matrix)	svg
5	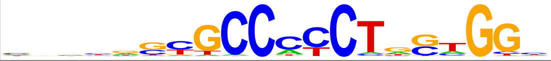	BORIS(Zf)/K562-CTCFL-ChIP-Seq(GSE32465)/Homer	1e-6	-1.506e+01	0.0001	178.0	20.11%	6207.8	13.94%	motif file (matrix)	svg
6	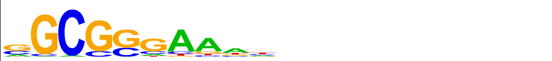	E2F4(E2F)/K562-E2F4-ChIP-Seq(GSE31477)/Homer	1e-5	-1.329e+01	0.0001	230.0	25.99%	8697.0	19.52%	motif file (matrix)	svg
7	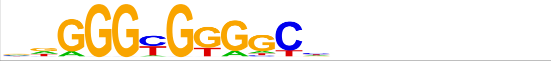	KLF1(Zf)/HUDEP2-KLF1-CutnRun(GSE136251)/Homer	1e-5	-1.158e+01	0.0006	456.0	51.53%	19734.4	44.30%	motif file (matrix)	svg
8	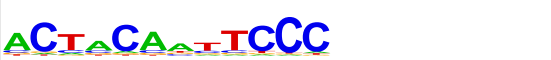	GFY(?)/Promoter/Homer	1e-3	-9.127e+00	0.0060	53.0	5.99%	1539.5	3.46%	motif file (matrix)	svg
9	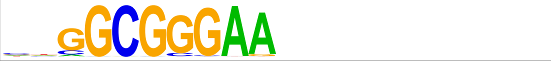	E2F1(E2F)/Hela-E2F1-ChIP-Seq(GSE22478)/Homer	1e-3	-6.965e+00	0.0462	120.0	13.56%	4551.7	10.22%	motif file (matrix)	svg
10	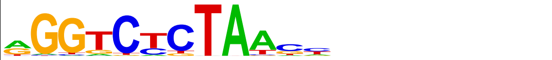	PRDM14(Zf)/H1-PRDM14-ChIP-Seq(GSE22767)/Homer	1e-2	-6.883e+00	0.0462	195.0	22.03%	7977.8	17.91%	motif file (matrix)	svg
11	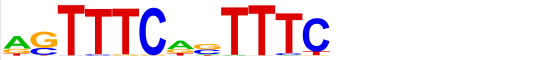	ISRE(IRF)/ThioMac-LPS-Expression(GSE23622)/Homer	1e-2	-6.075e+00	0.0919	39.0	4.41%	1199.9	2.69%	motif file (matrix)	svg
12	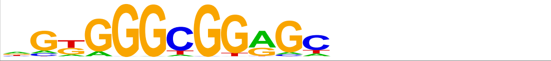	Sp5(Zf)/mES-Sp5.Flag-ChIP-Seq(GSE72989)/Homer	1e-2	-6.017e+00	0.0919	467.0	52.77%	21375.8	47.98%	motif file (matrix)	svg
13	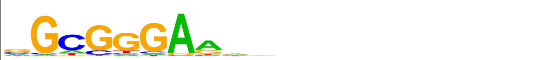	E2F6(E2F)/Hela-E2F6-ChIP-Seq(GSE31477)/Homer	1e-2	-5.804e+00	0.1020	247.0	27.91%	10628.0	23.86%	motif file (matrix)	svg
