# Supplementary material for: Chromatin accessibility and H3K9me3 landscapes reveal long-term epigenetic effects of fetal-neonatal iron deficiency in rat hippocampus
Source: BMC Genomics. 2024 Mar 21;25:301. doi: 10.1186/s12864-024-10230-4 (PMC10956188; doi:10.1186/s12864-024-10230-4)
Supplement: Supplementary file 4 — Supplementary Material 4. [file 12864_2024_10230_MOESM4_ESM.pdf]

S4 Motifs identified by ATAC-seq and HOMER analysis in the promoter region with increased accessibility in the FIDch group.

Homer Known Motif Enrichment Results (IDch_uppm.tab_genome)

Homer *de novo* Motif Results
Gene Ontology Enrichment Results
Known Motif Enrichment Results (txt file)
Total Target Sequences = 2216, Total Background Sequences = 36974

Rank	Motif	Name	P-value	log P-pvalue	q-value (Benjamini)	# Target Sequences with Motif	% of Targets Sequences with Motif	# Background Sequences with Motif	% of Background Sequences with Motif	Motif File	SVG
1	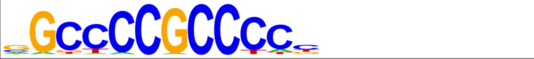	Sp1(Zf)/Promoter/Homer	1e-29	-6.751e+01	0.0000	1100.0	49.64%	13965.2	37.77%	motif file (matrix)	svg
2	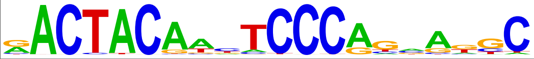	Ronin(THAP)/ES-Thap11-ChIP-Seq(GSE51522)/Homer	1e-12	-2.882e+01	0.0000	121.0	5.46%	979.5	2.65%	motif file (matrix)	svg
3	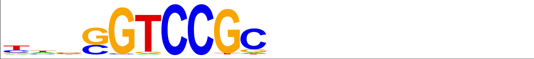	HINFP(Zf)/K562-HINFP.eGFP-ChIP-Seq(Encode)/Homer	1e-11	-2.707e+01	0.0000	1004.0	45.31%	14063.6	38.04%	motif file (matrix)	svg
4	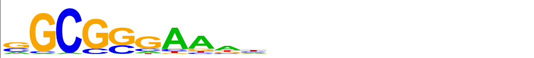	E2F4(E2F)/K562-E2F4-ChIP-Seq(GSE31477)/Homer	1e-11	-2.609e+01	0.0000	1132.0	51.08%	16210.8	43.85%	motif file (matrix)	svg
5	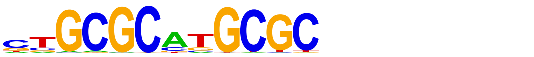	NRF1(NRF)/MCF7-NRF1-ChIP-Seq(Unpublished)/Homer	1e-11	-2.599e+01	0.0000	543.0	24.50%	6896.3	18.65%	motif file (matrix)	svg
6	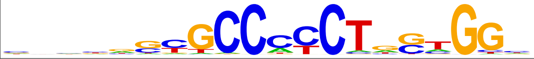	BORIS(Zf)/K562-CTCFL-ChIP-Seq(GSE32465)/Homer	1e-9	-2.118e+01	0.0000	629.0	28.38%	8434.6	22.81%	motif file (matrix)	svg
7	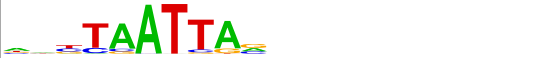	Lhx3(Homeobox)/Neuron-Lhx3-ChIP-Seq(GSE31456)/Homer	1e-8	-1.993e+01	0.0000	1272.0	57.40%	18915.4	51.16%	motif file (matrix)	svg
8	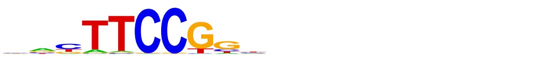	Elk1(ETS)/Hela-Elk1-ChIP-Seq(GSE31477)/Homer	1e-8	-1.981e+01	0.0000	1123.0	50.68%	16438.1	44.46%	motif file (matrix)	svg
9	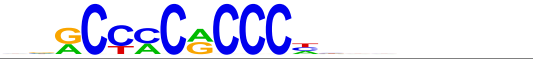	KLF3(Zf)/MEF-Klf3-ChIP-Seq(GSE44748)/Homer	1e-8	-1.976e+01	0.0000	1203.0	54.29%	17770.1	48.06%	motif file (matrix)	svg
10	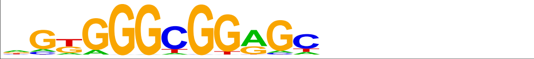	Sp5(Zf)/mES-Sp5.Flag-ChIP-Seq(GSE72989)/Homer	1e-8	-1.973e+01	0.0000	1736.0	78.34%	26964.8	72.93%	motif file (matrix)	svg
11	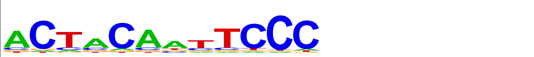	GFY(?)/Promoter/Homer	1e-8	-1.967e+01	0.0000	172.0	7.76%	1799.1	4.87%	motif file (matrix)	svg
12	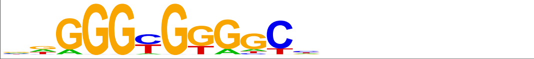	KLF1(Zf)/HUDEP2-KLF1-CutnRun(GSE136251)/Homer	1e-8	-1.943e+01	0.0000	1624.0	73.29%	24995.7	67.61%	motif file (matrix)	svg
13	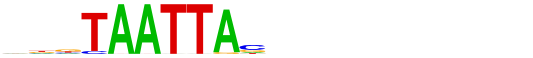	Dlx3(Homeobox)/Kerainocytes-Dlx3-ChIP-Seq(GSE89884)/Homer	1e-7	-1.818e+01	0.0000	614.0	27.71%	8362.4	22.62%	motif file (matrix)	svg
14	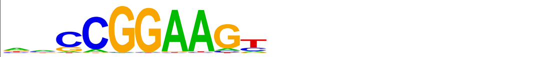	ELF1(ETS)/Jurkat-ELF1-ChIP-Seq(SRA014231)/Homer	1e-7	-1.699e+01	0.0000	1025.0	46.25%	15014.0	40.61%	motif file (matrix)	svg
15	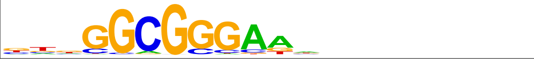	E2F3(E2F)/MEF-E2F3-ChIP-Seq(GSE71376)/Homer	1e-7	-1.684e+01	0.0000	1328.0	59.93%	20070.9	54.29%	motif file (matrix)	svg
16	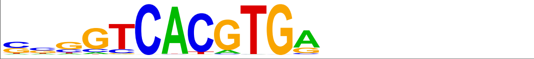	E-box(bHLH)/Promoter/Homer	1e-7	-1.658e+01	0.0000	198.0	8.94%	2242.3	6.07%	motif file (matrix)	svg
17	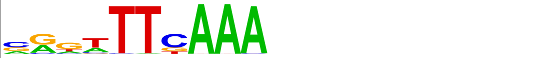	CHR(?)/Hela-CellCycle-Expression/Homer	1e-7	-1.648e+01	0.0000	679.0	30.64%	9480.5	25.64%	motif file (matrix)	svg
18	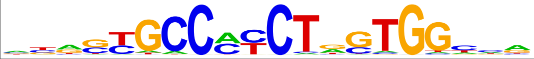	CTCF(Zf)/CD4+-CTCF-ChIP-Seq(Barski_et_al.)/Homer	1e-7	-1.627e+01	0.0000	340.0	15.34%	4295.3	11.62%	motif file (matrix)	svg
19	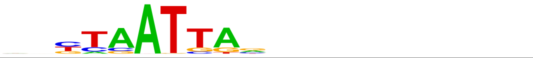	Lhx1(Homeobox)/EmbryoCarcinoma-Lhx1-ChIP-Seq(GSE70957)/Homer	1e-5	-1.364e+01	0.0000	927.0	41.83%	13655.5	36.94%	motif file (matrix)	svg
20	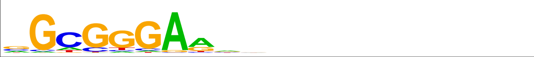	E2F6(E2F)/Hela-E2F6-ChIP-Seq(GSE31477)/Homer	1e-5	-1.350e+01	0.0000	1200.0	54.15%	18171.1	49.15%	motif file (matrix)	svg
21	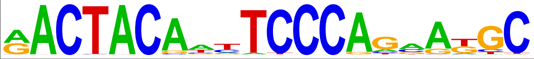	GFY-Staf(?,Zf)/Promoter/Homer	1e-5	-1.336e+01	0.0000	165.0	7.45%	1889.7	5.11%	motif file (matrix)	svg
22	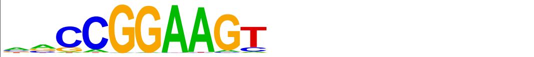	ETS(ETS)/Promoter/Homer	1e-5	-1.322e+01	0.0000	668.0	30.14%	9520.2	25.75%	motif file (matrix)	svg
23	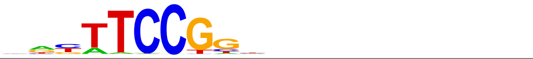	Elk4(ETS)/Hela-Elk4-ChIP-Seq(GSE31477)/Homer	1e-5	-1.302e+01	0.0000	1134.0	51.17%	17111.4	46.28%	motif file (matrix)	svg
24	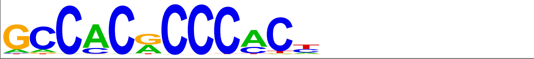	Klf9(Zf)/GBM-Klf9-ChIP-Seq(GSE62211)/Homer	1e-5	-1.301e+01	0.0000	999.0	45.08%	14882.4	40.25%	motif file (matrix)	svg
25	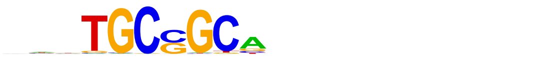	Zfp57(Zf)/H1-ZFP57.HA-ChIP-Seq(GSE115387)/Homer	1e-5	-1.293e+01	0.0000	941.0	42.46%	13939.6	37.70%	motif file (matrix)	svg
26	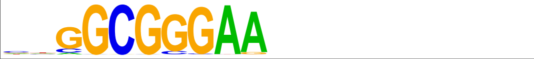	E2F1(E2F)/Hela-E2F1-ChIP-Seq(GSE22478)/Homer	1e-5	-1.263e+01	0.0001	722.0	32.58%	10422.8	28.19%	motif file (matrix)	svg
27	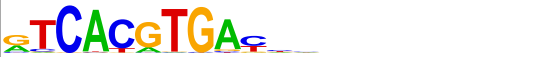	TFE3(bHLH)/MEF-TFE3-ChIP-Seq(GSE75757)/Homer	1e-5	-1.258e+01	0.0001	157.0	7.08%	1804.8	4.88%	motif file (matrix)	svg
28	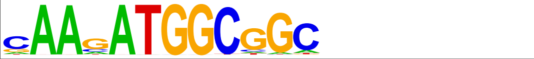	YY1(Zf)/Promoter/Homer	1e-5	-1.154e+01	0.0002	219.0	9.88%	2729.1	7.38%	motif file (matrix)	svg

29	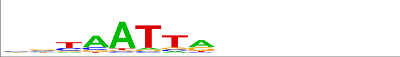	DLX5(Homeobox)/BasalGanglia-Dlx5-ChIP-seq(GSE124936)/Homer	1e-4	-1.091e+01	0.0003	692.0	31.23%	10074.2	27.25%	motif file (matrix)	svg
30	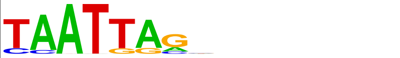	Lhx2(Homeobox)/HFSC-Lhx2-ChIP-Seq(GSE48068)/Homer	1e-4	-1.007e+01	0.0006	894.0	40.34%	13412.2	36.28%	motif file (matrix)	svg
31	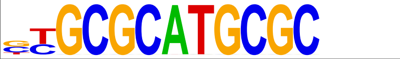	NRF(NRF)/Promoter/Homer	1e-4	-9.956e+00	0.0007	523.0	23.60%	7464.1	20.19%	motif file (matrix)	svg
32	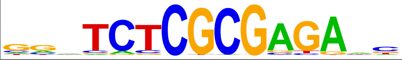	ZBTB33(Zf)/GM12878-ZBTB33-ChIP-Seq(GSE32465)/Homer	1e-4	-9.793e+00	0.0008	171.0	7.72%	2108.1	5.70%	motif file (matrix)	svg
33	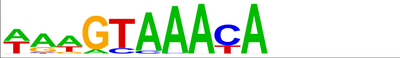	FOXA1(Forkhead)/MCF7-FOXA1-ChIP-Seq(GSE26831)/Homer	1e-4	-9.235e+00	0.0013	901.0	40.66%	13604.9	36.80%	motif file (matrix)	svg
34	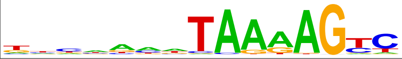	MYNN(Zf)/HEK293-MYNN.eGFP-ChIP-Seq(Encode)/Homer	1e-3	-9.131e+00	0.0014	286.0	12.91%	3849.8	10.41%	motif file (matrix)	svg
35	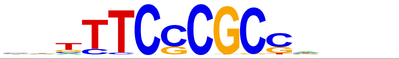	E2F7(E2F)/Hela-E2F7-ChIP-Seq(GSE32673)/Homer	1e-3	-9.021e+00	0.0015	397.0	17.92%	5561.5	15.04%	motif file (matrix)	svg
36	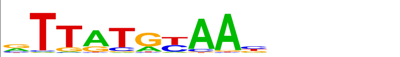	HLF(bZIP)/HSC-HLF.Flag-ChIP-Seq(GSE69817)/Homer	1e-3	-8.727e+00	0.0020	687.0	31.00%	10179.0	27.53%	motif file (matrix)	svg
37	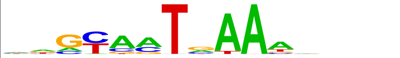	Hoxd12(Homeobox)/ChickenMSG-Hoxd12.Flag-ChIP-Seq(GSE86088)/Homer	1e-3	-8.651e+00	0.0021	1320.0	59.57%	20624.7	55.79%	motif file (matrix)	svg
38	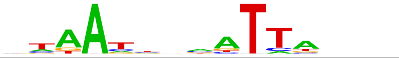	Prop1(Homeobox)/GHFT1-PROP1.biotin-ChIP-Seq(GSE77302)/Homer	1e-3	-8.590e+00	0.0022	500.0	22.56%	7207.7	19.50%	motif file (matrix)	svg
39	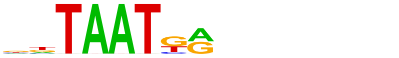	Nkx6.1(Homeobox)/Islet-Nkx6.1-ChIP-Seq(GSE40975)/Homer	1e-3	-8.267e+00	0.0029	1567.0	70.71%	24866.4	67.26%	motif file (matrix)	svg
40	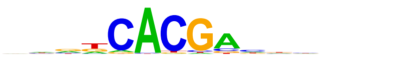	Npas4(bHLH)/Neuron-Npas4-ChIP-Seq(GSE127793)/Homer	1e-3	-8.095e+00	0.0034	1180.0	53.25%	18333.2	49.59%	motif file (matrix)	svg
41	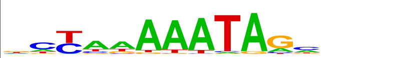	Mef2c(MADS)/GM12878-Mef2c-ChIP-Seq(GSE32465)/Homer	1e-3	-7.837e+00	0.0042	419.0	18.91%	5992.2	16.21%	motif file (matrix)	svg
42	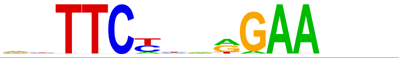	STAT6(Stat)/CD4-Stat6-ChIP-Seq(GSE22104)/Homer	1e-3	-7.665e+00	0.0049	572.0	25.81%	8431.0	22.80%	motif file (matrix)	svg
43	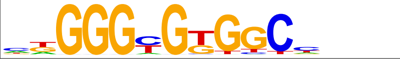	KLF6(Zf)/PDAC-KLF6-ChIP-Seq(GSE64557)/Homer	1e-3	-7.645e+00	0.0049	1678.0	75.72%	26843.9	72.61%	motif file (matrix)	svg
44	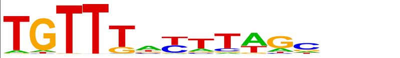	FoxD3(forkhead)/ZebrafishEmbryo-Foxd3.biotin-ChIP-seq(GSE106676)/Homer	1e-3	-7.286e+00	0.0068	833.0	37.59%	12688.3	34.32%	motif file (matrix)	svg
45	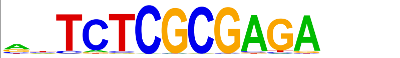	GFX(?)/Promoter/Homer	1e-3	-7.107e+00	0.0080	53.0	2.39%	553.8	1.50%	motif file (matrix)	svg
46	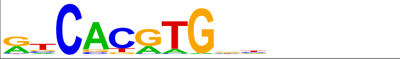	Usf2(bHLH)/C2C12-Usf2-ChIP-Seq(GSE36030)/Homer	1e-3	-7.076e+00	0.0081	439.0	19.81%	6369.7	17.23%	motif file (matrix)	svg
47	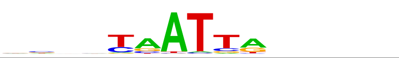	DLX1(Homeobox)/BasalGanglia-Dlx1-ChIP-seq(GSE124936)/Homer	1e-2	-6.881e+00	0.0096	1032.0	46.57%	16007.5	43.30%	motif file (matrix)	svg
48	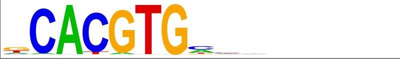	bHLHE40(bHLH)/HepG2-BHLHE40-ChIP-Seq(GSE31477)/Homer	1e-2	-6.581e+00	0.0127	507.0	22.88%	7493.8	20.27%	motif file (matrix)	svg
49	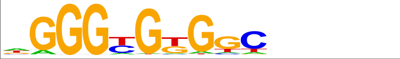	KLF5(Zf)/LoVo-KLF5-ChIP-Seq(GSE49402)/Homer	1e-2	-6.273e+00	0.0169	1797.0	81.09%	29051.3	78.58%	motif file (matrix)	svg
50	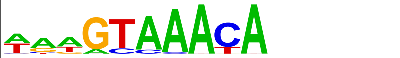	FOXA1(Forkhead)/LNCAP-FOXA1-ChIP-Seq(GSE27824)/Homer	1e-2	-6.232e+00	0.0173	1023.0	46.16%	15935.5	43.10%	motif file (matrix)	svg
51	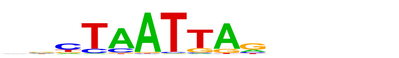	LHX9(Homeobox)/Hct116-LHX9.V5-ChIP-Seq(GSE116822)/Homer	1e-2	-6.221e+00	0.0173	1138.0	51.35%	17847.1	48.27%	motif file (matrix)	svg
52	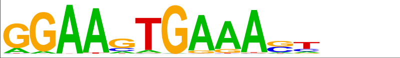	PU.1:IRF8(ETS:IRF)/pDC-Irf8-ChIP-Seq(GSE66899)/Homer	1e-2	-6.167e+00	0.0177	249.0	11.24%	3474.5	9.40%	motif file (matrix)	svg
53	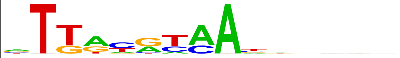	NFIL3(bZIP)/HepG2-NFIL3-ChIP-Seq(Encode)/Homer	1e-2	-6.102e+00	0.0186	559.0	25.23%	8373.1	22.65%	motif file (matrix)	svg
54	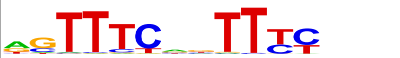	IRF3(IRF)/BMDM-Irf3-ChIP-Seq(GSE67343)/Homer	1e-2	-5.798e+00	0.0247	387.0	17.46%	5660.4	15.31%	motif file (matrix)	svg
55	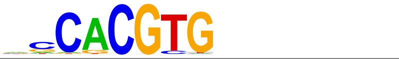	c-Myc(bHLH)/LNCAP-cMyc-ChIP-Seq(Unpublished)/Homer	1e-2	-5.795e+00	0.0247	831.0	37.50%	12825.4	34.69%	motif file (matrix)	svg
56	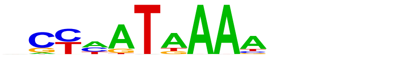	Hoxd13(Homeobox)/ChickenMSG-Hoxd13.Flag-ChIP-Seq(GSE86088)/Homer	1e-2	-5.663e+00	0.0273	1200.0	54.15%	18953.6	51.27%	motif file (matrix)	svg
57	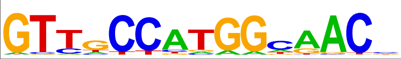	Rfx2(HTH)/LoVo-RFX2-ChIP-Seq(GSE49402)/Homer	1e-2	-5.539e+00	0.0304	173.0	7.81%	2354.4	6.37%	motif file (matrix)	svg
58	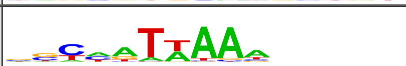	Unknown(Homeobox)/Limb-p300-ChIP-Seq/Homer	1e-2	-5.456e+00	0.0324	612.0	27.62%	9298.6	25.15%	motif file (matrix)	svg
59	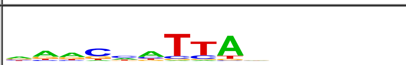	Barx1(Homeobox)/Stomach-Barx1.3xFlag-ChIP-Seq(GSE69483)/Homer	1e-2	-5.456e+00	0.0324	446.0	20.13%	6630.0	17.93%	motif file (matrix)	svg
60	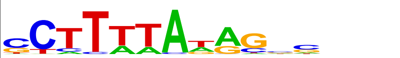	TATA-Box(TBP)/Promoter/Homer	1e-2	-5.420e+00	0.0325	1252.0	56.50%	19856.0	53.71%	motif file (matrix)	svg

61	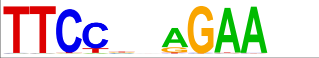	STAT6(Stat)/Macrophage-Stat6-ChIP-Seq(GSE38377)/Homer	1e-2	-5.286e+00	0.0365	579.0	26.13%	8784.4	23.76%	motif file (matrix)	svg
62	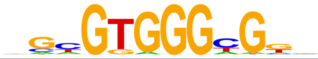	Egr2(Zf)/Thymocytes-Egr2-ChIP-Seq(GSE34254)/Homer	1e-2	-5.220e+00	0.0384	527.0	23.78%	7954.0	21.51%	motif file (matrix)	svg
63	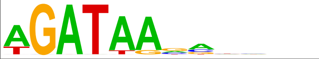	TRPS1(Zf)/MCF7-TRPS1-ChIP-Seq(GSE107013)/Homer	1e-2	-5.184e+00	0.0392	1417.0	63.94%	22666.8	61.31%	motif file (matrix)	svg
64	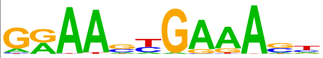	IRF8(IRF)/BMDM-IRF8-ChIP-Seq(GSE77884)/Homer	1e-2	-5.081e+00	0.0427	410.0	18.50%	6094.2	16.48%	motif file (matrix)	svg
65	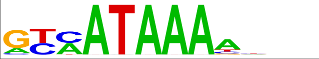	Cdx2(Homeobox)/mES-Cdx2-ChIP-Seq(GSE14586)/Homer	1e-2	-5.051e+00	0.0434	614.0	27.71%	9378.7	25.37%	motif file (matrix)	svg
66	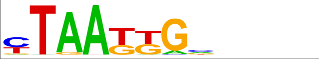	Isl1(Homeobox)/Neuron-Isl1-ChIP-Seq(GSE31456)/Homer	1e-2	-4.919e+00	0.0487	1505.0	67.92%	24193.9	65.44%	motif file (matrix)	svg
67	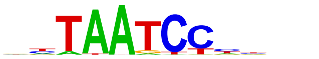	Otx2(Homeobox)/EpiLC-Otx2-ChIP-Seq(GSE56098)/Homer	1e-2	-4.863e+00	0.0508	815.0	36.78%	12682.4	34.30%	motif file (matrix)	svg
68	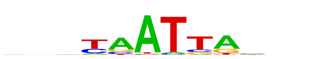	DLX2(Homeobox)/BasalGanglia-Dlx2-ChIP-seq(GSE124936)/Homer	1e-2	-4.851e+00	0.0508	1116.0	50.36%	17662.7	47.77%	motif file (matrix)	svg
69	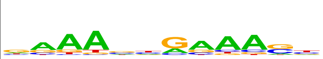	IRF2(IRF)/Erythroblas-IRF2-ChIP-Seq(GSE36985)/Homer	1e-2	-4.734e+00	0.0560	146.0	6.59%	1995.1	5.40%	motif file (matrix)	svg
70	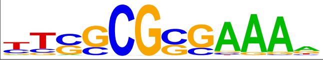	E2F(E2F)/Hela-CellCycle-Expression/Homer	1e-2	-4.719e+00	0.0561	163.0	7.36%	2255.4	6.10%	motif file (matrix)	svg
