# Supplementary material for: Chromatin accessibility and H3K9me3 landscapes reveal long-term epigenetic effects of fetal-neonatal iron deficiency in rat hippocampus
Source: BMC Genomics. 2024 Mar 21;25:301. doi: 10.1186/s12864-024-10230-4 (PMC10956188; doi:10.1186/s12864-024-10230-4)
Supplement: Supplementary file 5 — Supplementary Material 5. [file 12864_2024_10230_MOESM5_ESM.pdf]

S5 Motifs identified by ATAC-seq and HOMER analysis in the promoter region with increased accessibility in the ISch group.

Homer Known Motif Enrichment Results (ISch_uppm.tab_genome)

Homer *de novo* Motif Results
Gene Ontology Enrichment Results
Known Motif Enrichment Results (txt file)
Total Target Sequences = 1059, Total Background Sequences = 42108

Rank	Motif	Name	P-value	log P-pvalue	q-value (Benjamini)	# Target Sequences with Motif	% of Targets Sequences with Motif	# Background Sequences with Motif	% of Background Sequences with Motif	Motif File	SVG
1	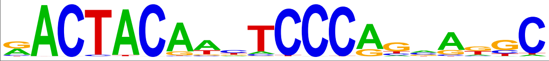	Ronin(THAP)/ES-Thap11-ChIP-Seq(GSE51522)/Homer	1e-39	-9.122e+01	0.0000	94.0	8.88%	665.3	1.58%	motif file (matrix)	svg
2	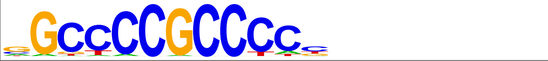	Sp1(Zf)/Promoter/Homer	1e-39	-9.019e+01	0.0000	333.0	31.47%	6446.3	15.26%	motif file (matrix)	svg
3	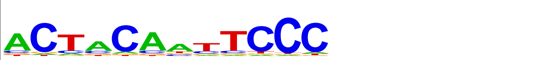	GFY(?)/Promoter/Homer	1e-18	-4.347e+01	0.0000	115.0	10.87%	1789.6	4.24%	motif file (matrix)	svg
4	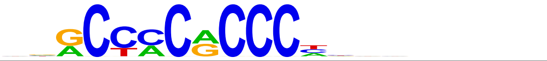	KLF3(Zf)/MEF-Klf3-ChIP-Seq(GSE44748)/Homer	1e-16	-3.778e+01	0.0000	456.0	43.10%	13038.1	30.87%	motif file (matrix)	svg
5	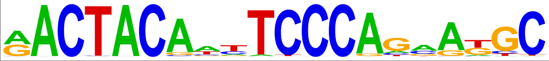	GFY-Staf(?,Zf)/Promoter/Homer	1e-15	-3.607e+01	0.0000	129.0	12.19%	2358.5	5.58%	motif file (matrix)	svg
6	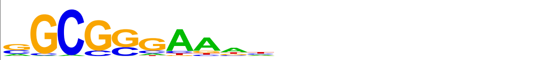	E2F4(E2F)/K562-E2F4-ChIP-Seq(GSE31477)/Homer	1e-13	-3.207e+01	0.0000	341.0	32.23%	9297.1	22.01%	motif file (matrix)	svg
7	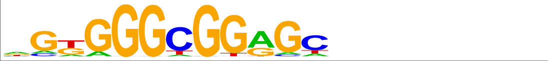	Sp5(Zf)/mES-Sp5.Flag-ChIP-Seq(GSE72989)/Homer	1e-12	-2.839e+01	0.0000	675.0	63.80%	22334.1	52.88%	motif file (matrix)	svg
8	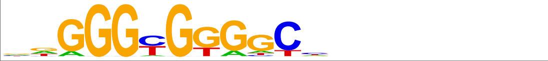	KLF1(Zf)/HUDEP2-KLF1-CutnRun(GSE136251)/Homer	1e-11	-2.640e+01	0.0000	630.0	59.55%	20685.5	48.98%	motif file (matrix)	svg
9	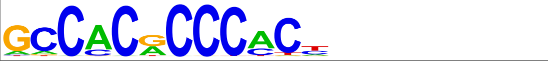	Klf9(Zf)/GBM-Klf9-ChIP-Seq(GSE62211)/Homer	1e-10	-2.463e+01	0.0000	368.0	34.78%	10805.7	25.59%	motif file (matrix)	svg
10	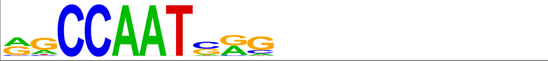	NFY(CCAAT)/Promoter/Homer	1e-9	-2.200e+01	0.0000	549.0	51.89%	17889.7	42.36%	motif file (matrix)	svg
11	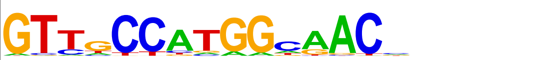	Rfx2(HTH)/LoVo-RFX2-ChIP-Seq(GSE49402)/Homer	1e-8	-1.881e+01	0.0000	108.0	10.21%	2410.2	5.71%	motif file (matrix)	svg
12	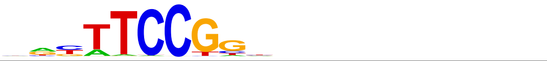	Elk4(ETS)/Hela-Elk4-ChIP-Seq(GSE31477)/Homer	1e-7	-1.784e+01	0.0000	500.0	47.26%	16415.9	38.87%	motif file (matrix)	svg
13	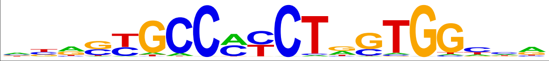	CTCF(Zf)/CD4+-CTCF-ChIP-Seq(Barski_et_al.)/Homer	1e-7	-1.676e+01	0.0000	190.0	17.96%	5173.2	12.25%	motif file (matrix)	svg
14	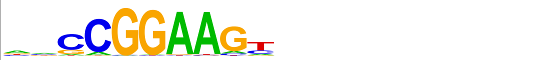	ELF1(ETS)/Jurkat-ELF1-ChIP-Seq(SRA014231)/Homer	1e-7	-1.624e+01	0.0000	435.0	41.12%	14096.7	33.38%	motif file (matrix)	svg
15	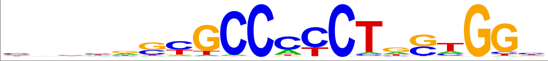	BORIS(Zf)/K562-CTCFL-ChIP-Seq(GSE32465)/Homer	1e-6	-1.562e+01	0.0000	239.0	22.59%	6957.7	16.47%	motif file (matrix)	svg
16	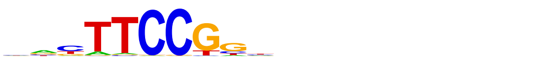	Elk1(ETS)/Hela-Elk1-ChIP-Seq(GSE31477)/Homer	1e-6	-1.468e+01	0.0000	465.0	43.95%	15427.5	36.53%	motif file (matrix)	svg
17	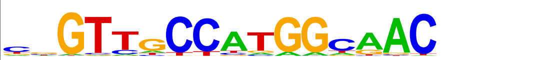	RFX(HTH)/K562-RFX3-ChIP-Seq(SRA012198)/Homer	1e-6	-1.392e+01	0.0000	93.0	8.79%	2195.8	5.20%	motif file (matrix)	svg
18	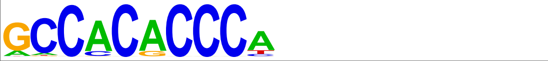	Klf4(Zf)/mES-Klf4-ChIP-Seq(GSE11431)/Homer	1e-5	-1.239e+01	0.0001	323.0	30.53%	10327.9	24.45%	motif file (matrix)	svg
19	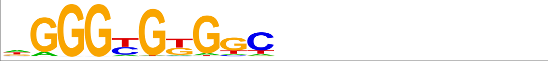	KLF5(Zf)/LoVo-KLF5-ChIP-Seq(GSE49402)/Homer	1e-5	-1.235e+01	0.0001	727.0	68.71%	26233.9	62.12%	motif file (matrix)	svg
20	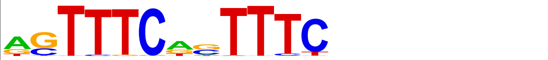	ISRE(IRF)/ThioMac-LPS-Expression(GSE23622)/Homer	1e-4	-1.036e+01	0.0007	57.0	5.39%	1278.7	3.03%	motif file (matrix)	svg
21	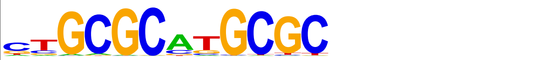	NRF1(NRF)/MCF7-NRF1-ChIP-Seq(Unpublished)/Homer	1e-4	-9.873e+00	0.0011	118.0	11.15%	3268.3	7.74%	motif file (matrix)	svg
22	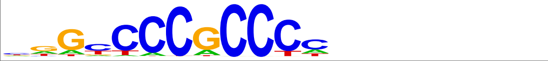	Sp2(Zf)/HEK293-Sp2.eGFP-ChIP-Seq(Encode)/Homer	1e-4	-9.796e+00	0.0011	781.0	73.82%	28858.8	68.33%	motif file (matrix)	svg
23	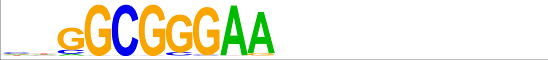	E2F1(E2F)/Hela-E2F1-ChIP-Seq(GSE22478)/Homer	1e-4	-9.659e+00	0.0012	163.0	15.41%	4836.7	11.45%	motif file (matrix)	svg
24	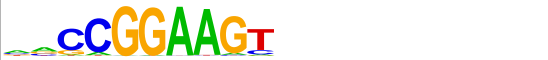	ETS(ETS)/Promoter/Homer	1e-4	-9.634e+00	0.0012	291.0	27.50%	9477.2	22.44%	motif file (matrix)	svg
25	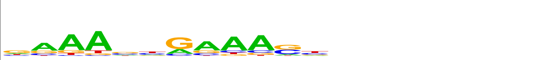	IRF2(IRF)/Erythroblas-IRF2-ChIP-Seq(GSE36985)/Homer	1e-4	-9.237e+00	0.0017	91.0	8.60%	2417.5	5.72%	motif file (matrix)	svg
26	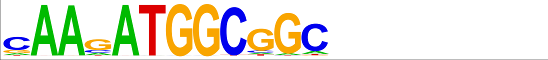	YY1(Zf)/Promoter/Homer	1e-3	-9.141e+00	0.0018	80.0	7.56%	2065.4	4.89%	motif file (matrix)	svg
27	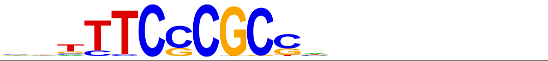	E2F7(E2F)/Hela-E2F7-ChIP-Seq(GSE32673)/Homer	1e-3	-8.434e+00	0.0035	94.0	8.88%	2576.4	6.10%	motif file (matrix)	svg

28	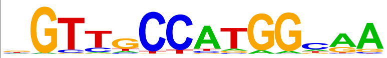	Rfx1(HTH)/NPC-H3K4me1-ChIP-Seq(GSE16256)/Homer	1e-3	-8.147e+00	0.0045	167.0	15.78%	5134.9	12.16%	motif file (matrix)	svg
29	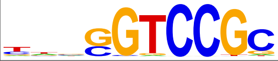	HINFP(Zf)/K562-HINFP.eGFP-ChIP-Seq(Encode)/Homer	1e-3	-7.426e+00	0.0090	205.0	19.38%	6594.8	15.62%	motif file (matrix)	svg
30	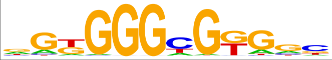	KLF14(Zf)/HEK293-KLF14.GFP-ChIP-Seq(GSE58341)/Homer	1e-3	-7.075e+00	0.0124	836.0	79.02%	31613.3	74.86%	motif file (matrix)	svg
31	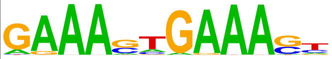	IRF1(IRF)/PBMC-IRF1-ChIP-Seq(GSE43036)/Homer	1e-2	-6.797e+00	0.0159	109.0	10.30%	3231.3	7.65%	motif file (matrix)	svg
32	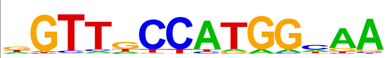	X-box(HTH)/NPC-H3K4me1-ChIP-Seq(GSE16256)/Homer	1e-2	-6.108e+00	0.0306	93.0	8.79%	2743.2	6.50%	motif file (matrix)	svg
33	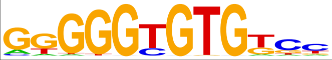	KLF10(Zf)/HEK293-KLF10.GFP-ChIP-Seq(GSE58341)/Homer	1e-2	-5.769e+00	0.0417	501.0	47.35%	18215.5	43.13%	motif file (matrix)	svg
34	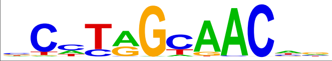	Rfx5(HTH)/GM12878-Rfx5-ChIP-Seq(GSE31477)/Homer	1e-2	-5.528e+00	0.0514	296.0	27.98%	10296.3	24.38%	motif file (matrix)	svg
35	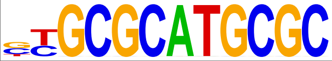	NRF(NRF)/Promoter/Homer	1e-2	-5.313e+00	0.0620	149.0	14.08%	4833.4	11.44%	motif file (matrix)	svg
36	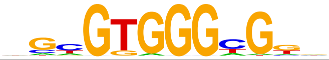	Egr2(Zf)/Thymocytes-Egr2-ChIP-Seq(GSE34254)/Homer	1e-2	-5.230e+00	0.0654	141.0	13.33%	4553.4	10.78%	motif file (matrix)	svg
37	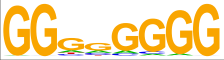	Maz(Zf)/HepG2-Maz-ChIP-Seq(GSE31477)/Homer	1e-2	-5.110e+00	0.0718	719.0	67.96%	27129.7	64.24%	motif file (matrix)	svg
38	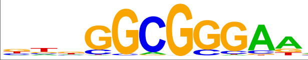	E2F3(E2F)/MEF-E2F3-ChIP-Seq(GSE71376)/Homer	1e-2	-4.670e+00	0.1086	375.0	35.44%	13515.2	32.00%	motif file (matrix)	svg
39	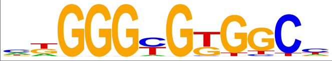	KLF6(Zf)/PDAC-KLF6-ChIP-Seq(GSE64557)/Homer	1e-2	-4.621e+00	0.1110	629.0	59.45%	23588.4	55.85%	motif file (matrix)	svg
