# Supplementary material for: Chromatin accessibility and H3K9me3 landscapes reveal long-term epigenetic effects of fetal-neonatal iron deficiency in rat hippocampus
Source: BMC Genomics. 2024 Mar 21;25:301. doi: 10.1186/s12864-024-10230-4 (PMC10956188; doi:10.1186/s12864-024-10230-4)
Supplement: Supplementary file 6 — Supplementary Material 6. [file 12864_2024_10230_MOESM6_ESM.pdf]

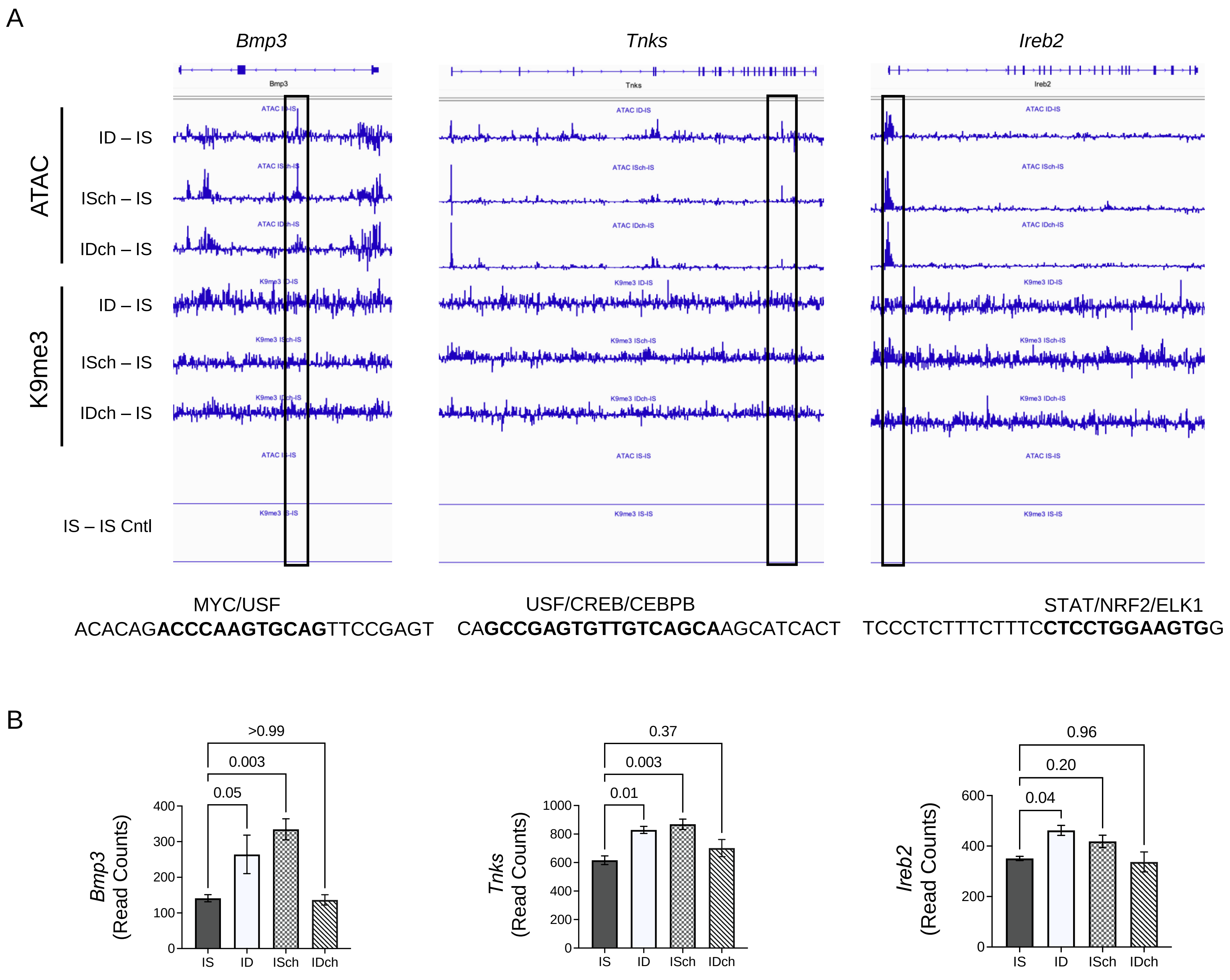

S6: IGV alignments of ATAC and H3K9me3 peaks. (A) Peaks aligned to *Bmp3*, *Tnks*, and *Ireb2* loci showing differential effects by iron deficiency and choline. Brackets depict regions with differential peaks among ID, ISch, and IDch groups and potential enhancers with specific transcription factor binding sites. (B) Graphs showing transcript counts of *Bmp3*, *Tnks*, and *Ireb2* generated from RNAseq data. Values are mean +/- SEM, 2-tails t-test.
